# Age-dependent sex differences in cardiometabolic risk factors

**DOI:** 10.1038/s44161-022-00131-8

**Published:** 2022-09-12

**Authors:** Daria V. Zhernakova, Trishla Sinha, Sergio Andreu-Sánchez, Jelmer R. Prins, Alexander Kurilshikov, Jan-Willem Balder, Serena Sanna, Lifelines Cohort Study, Lude Franke, Jan A. Kuivenhoven, Alexandra Zhernakova, Jingyuan Fu

**Affiliations:** 1 Department of Genetics, University Medical Center Groningen, University of Groningen, Groningen, the Netherlands; 2 Laboratory of Genomic Diversity, Center for Computer Technologies, ITMO University, Saint Petersburg, Russia; 3 Department of Pediatrics, University Medical Center Groningen, University of Groningen, Groningen, the Netherlands; 4 Department of Obstetrics and Gynecology, University Medical Center Groningen, University of Groningen, Groningen, the Netherlands; 5 Division of Heart and Lungs, Department of Cardiology, University Medical Center Utrecht, Utrecht University, Utrecht, the Netherlands; 6 Istituto di Ricerca Genetica e Biomedica (IRGB) del Consiglio Nazionale delle Ricerche (CNR), Monserrato, Italy

**Keywords:** Cardiovascular diseases, Biomarkers, Data integration

## Abstract

Cardiometabolic diseases (CMDs) are a major cause of mortality worldwide, yet men and women present remarkable differences in disease prognosis, onset and manifestation. Here we characterize how sex differences in cardiometabolic risk factors vary with age by examining 45 phenotypes and 6 lifestyle factors in 146,021 participants of the Dutch population cohort Lifelines. We show that sex differences are present in 71% of the studied phenotypes. For 31% of these phenotypes, the phenotypic difference between sexes is dependent on age. CMD risk factors show various patterns of age-related sex differences, ranging from no difference for phenotypes such as body mass index (BMI) to strong age-modified sex differences for lipid levels. We also identify lifestyle factors that influence phenotypes in a sex- and age-dependent manner. These results highlight the importance of taking age into account when studying sex differences in CMDs.

## Abstract (web summary)

With the use of the Lifelines cohort, Zhernakova and colleagues set out to characterize sex differences in cardiometabolic risk factors, metabolites and proteins in adults aged 20–80 years and find a strong age effect on sex differences in cardiometabolic disease risk factors and biomarkers.

## Main

CMDs represent one of the largest health burdens in modern society and exhibit strong sex differences in disease incidence, severity and treatment efficiency^1^. Although the prevalence of CMD is generally lower in women than in men, CMDs remain the major cause of death for both sexes. In addition, diagnosis and treatment strategies in women are suboptimal, and sex-tailored prevention and treatment strategies are lacking^2^. To develop these strategies, it is important to understand the mechanisms that underlie the sex differences in CMD. On the one hand, some risk factors are known to exert sex-dependent effects^3–6^. Heavy smoking, for example, doubles the risk of myocardial infarction in women compared with its impact in men^3^. On the other hand, many cross-sectional comparisons between men and women have also highlighted differences on molecular levels such as gene expression and metabolomics, which suggests sex-dependent effects in disease etiology and molecular pathways^7^. In addition, CMD is an age-related disorder, and its prevalence increases rapidly with age^8^. The process of aging also shows remarkable differences between men and women^9^. An important but sometimes overlooked property of sex differences is that sex differences are not static—they change with age^6,10^. For instance, myocardial infarction incidence increases with age, but women get myocardial infarction around 10 yr later than men^11^. It is therefore important to take sex, age and their interactions into account to better understand the interplay of risk factors and improve disease prevention and treatment.

Given the important roles of both age and sex in CMD incidence, we think that the sex-dependent etiology of CMD should be studied in the context of aging, which may be reflected by the sex-dependent, nonlinear progression of CMD risk factors, and molecular traits with age. Previous studies have highlighted such age-dependent sex differences for several groups of CMD-related phenotypes, including CMD incidence and mortality^10,12^, major risk factors^6^ and lipid levels^13–16^. However, to our knowledge, no large-scale, systematic analysis of age-dependent sex differences has been performed to date for all CMD risk factors and biomarkers, including lifestyle factors and metabolic and proteomic profile.

CMD involves interactions between multiple organs and organ systems^17^. As a consequence, a large number of phenotypes are associated with CMD risk, ranging from well-established risk factors such as blood pressure to those less directly linked to CMD such as blood cell proportions^18^, serum albumin^19^ or uric acid^20^ levels. For disease prevention and diagnosis purposes, it is important to know which of these risk factors are causal. However, inferring causality is difficult, so the list of causal risk factors is constantly changing. The major risk factors that are currently considered causal include total cholesterol, low-density lipoprotein (LDL) cholesterol and triglyceride levels^21–23^; blood pressure^24^; BMI^25^; smoking^26^; alcohol consumption^27^; and type 2 diabetes^28^. Some evidence of causality has also been observed for lifestyle factors such as diet quality^29^, physical activity^30^ or stress^31^. Several phenotypes such as fasting glucose or high-density lipoprotein (HDL) cholesterol levels were previously considered causal but have not been confirmed by later studies^32,33^. While most studies on age and sex differences in CMD concentrate on established risk factors, it would be useful to profile patterns of age-related sex differences for a large range of potential risk factors to provide a broad view of the effect of age and sex on CMD-related mechanisms.

Here we made use of detailed phenotype data available for the Lifelines cohort—a large population cohort from the northern part of the Netherlands comprising more than 167,000 individuals from the general population^34^. In this cohort, we characterized sex differences across age span, starting with a wide range of 51 phenotypes and thereafter focusing on CMD risk factors. We further zoomed in on age-dependent sex differences in the plasma levels of 231 metabolites and 92 CMD-related proteins in a subset of 1,440 individuals. We investigated the general profile of sex differences across age and whether these changes are gradual or occur at specific ages.

## Results

**Fig. 1:**
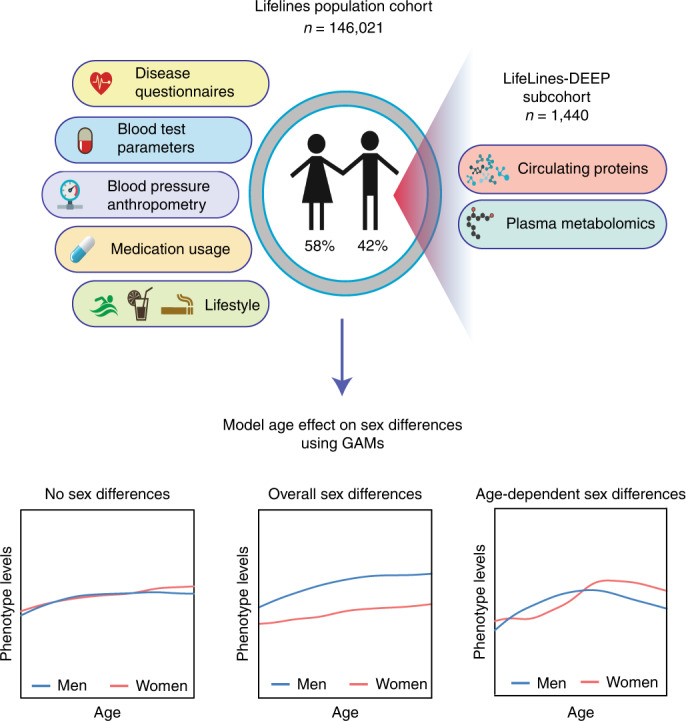
Study overview. This study involved a Dutch population-based cohort, Lifelines, consisting of 146,021 individuals for whom a large range of phenotype data have been profiled. For a subset of 1,440 individuals, there are additional data on serum proteomics and metabolomics. Using these data, we profiled age-related sex differences in a nonlinear way using GAMs.

**Extended Data Fig. 1:**
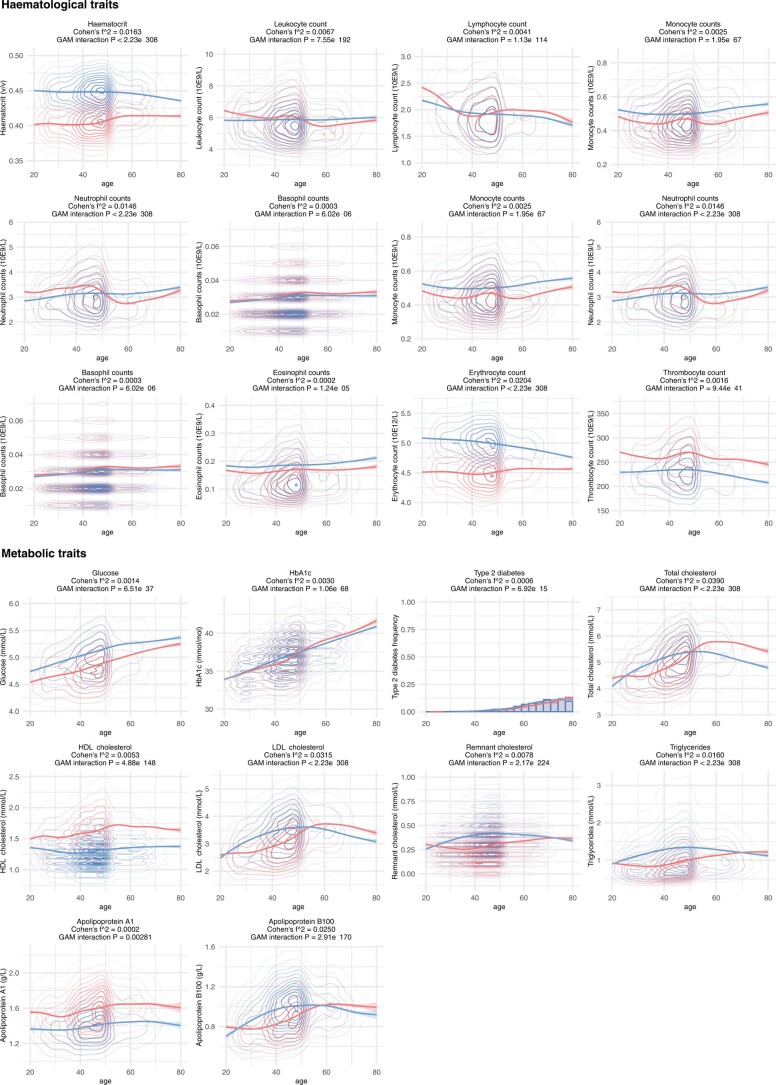
Age-dependent sex differences for hematological and metabolic phenotypes. The 16 For each phenotype, the plots show the density of observations for men (blue contours) and women (red contours) and fitted lines obtained using GAM: men (blue) and women (red). Confidence intervals (+/− 1.96 SE) around the lines are depicted in a more transparent hue. For binary phenotypes, colored bars represent the frequency of the phenotype in men (blue) and women (red). Cohen’s *f* ^2^ reflects the effect size of the age by sex interaction. GAM interaction P indicates the significance of the age by sex interaction term. Source data

**Extended Data Fig. 2:**
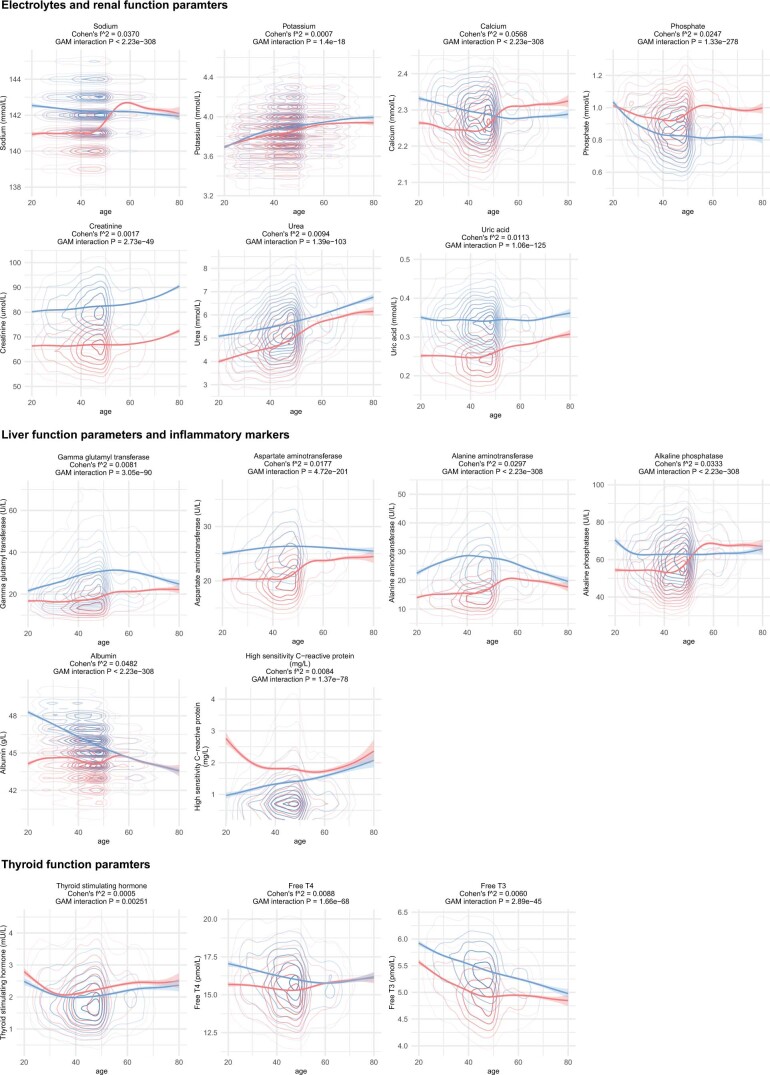
Age-dependent sex differences for electrolytes, kidney, liver and thyroid function paramters. For each phenotype, the plots show the density of observations for men (blue contours) and women (red contours) and fitted lines obtained using GAM: men (blue) and women (red). Confidence intervals (+/− 1.96 SE) around the lines are depicted in a more transparent hue. For binary phenotypes, colored bars represent the frequency of the phenotype in men (blue) and women (red). Cohen’s *f* ^2^ reflects the effect size of the age by sex interaction. GAM interaction P indicates the significance of the age by sex interaction term. Source data

**Extended Data Fig. 3:**
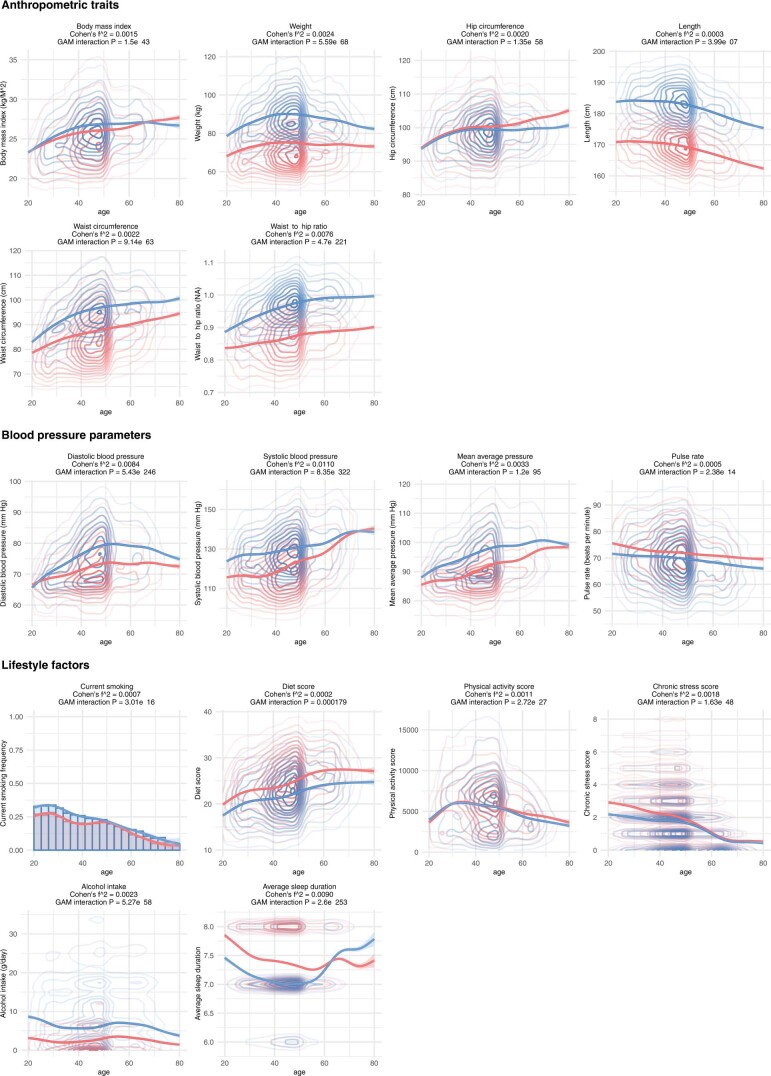
Age-dependent sex differences for anthropometric phenotypes and lifestyle factors. For each phenotype, the plots show the density of observations for men (blue contours) and women (red contours) and fitted lines obtained using GAM: men (blue) and women (red). Confidence intervals (+/− 1.96 SE) around the lines are depicted in a more transparent hue. For binary phenotypes, colored bars represent the frequency of the phenotype in men (blue) and women (red). Cohen’s *f* ^2^ reflects the effect size of the age by sex interaction. GAM interaction P indicates the significance of the age by sex interaction term. Source data

Our study involved 146,021 individuals (58% female) across an age span from 20 to 80 yr from the Dutch population-based cohort Lifelines. First, we assessed whether age effects on sex differences are widespread across a broad range of phenotypes that are commonly used as disease risk factors or biomarkers. We selected 51 phenotypes, including blood test parameters, anthropometric measurements, blood pressure and lifestyle factors (Supplementary Table 1 and Extended Data Figs. 1–3). Of these, 36 phenotypes (71%) exhibited linear sex differences over the whole age range (Supplementary Tables 2 and 3). However, we were specifically interested in phenotypes for which sex differences were not static with age but show an age by sex interaction (Fig. 1). Using a generalized additive model (GAM) approach, we found that all 51 phenotypes showed nonlinear age-dependent sex differences (Supplementary Table 4), and 16 of the 51 phenotypes (31%) showed a significant age by sex interaction with a considerable effect size (Bonferroni-adjusted interaction *P* < 0.05 and Cohen’s *f*^2^ ≥ 0.01; Methods) (Supplementary Tables 2 and 3 and Extended Data Figs. 1–3). Our results are consistent with age-related sex difference patterns previously reported for some of the studied phenotypes in participants of the UK Biobank cohort aged above 50 (ref. ^35^). The strongest effect was observed for plasma calcium levels (age by sex interaction *P*_inter_adj_ < 2.23 × 10^−308^, Cohen’s *f*^2^ = 0.06), which were lower in women than in men up to 45 yr of age and higher in women than in men after 55 yr (Extended Data Fig. 2), in line with previous reports^36^. This trend was still present after excluding calcium supplementation users (Supplementary Fig. 1).

Interestingly, the levels of six phenotypes with a significant age by sex interaction showed a similar pattern: they were higher in men before 50–60 yr of age, when they became higher in women. This pattern was observed for total and LDL cholesterol, apolipoprotein B100, sodium, calcium and alkaline phosphatase (Extended Data Figs. 1 and 2), whereas the reverse pattern was seen for leukocyte and neutrophil counts (Extended Data Fig. 1). For such phenotypes, sex difference estimations that average over the whole age span may give conflicting or insignificant results. For example, neutrophil count and total cholesterol levels do not show a significant sex difference across the whole age span but do show age-dependent sex differences (Supplementary Table 2), which further highlights the importance of studying differences between men and women and of taking age into account in these studies (Extended Data Figs. 1–3).

### Menopause is associated with age differences in clinical blood markers

**Fig. 2:**
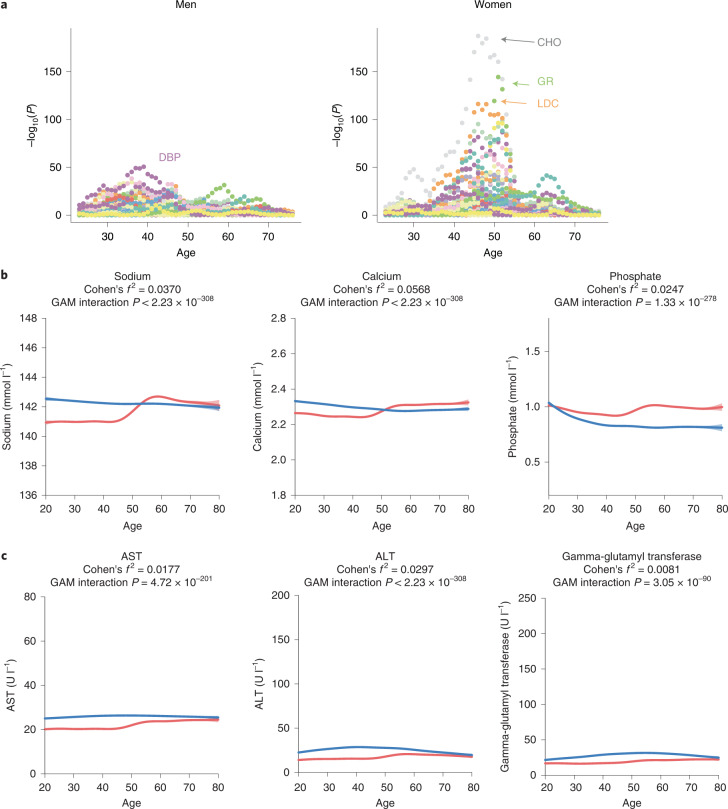
Multiple phenotypes exhibit rapid changes around the age of menopause in women compared with gradual changes over life in men. **a**, Age of the most significant differences in phenotype levels for each sex as determined by a sliding window *t*-test. Colors represent different phenotypes. For each phenotype for each year of age, dots represent −log_10_ of the *t*-test *P* value comparing mean phenotype levels before versus after this age. **b**,**c**, Age-dependent sex differences of blood test parameters: electrolytes (**b**) and liver function parameters (**c**). Lines correspond to the fitted GAMs in men (blue) and women (red). Confidence intervals (±1.96 s.e.m.) are plotted around the lines in a more transparent hue. Cohen’s *f*^2^ reflects the effect size of the age by sex interaction. GAM interaction *P* is the significance of the age by sex interaction term. CHO, total cholesterol; GR, neutrophil count; LDC, LDL cholesterol. Source data

Next, we zoomed in on the traits with age-dependent sex differences and searched for the turning point of aging when a strong difference in phenotype levels before and after was observed. Ten blood test parameters out of the 16 phenotypes with an age by sex interaction (62%) exhibited a sex-specific age pattern: the levels of these phenotypes were different in women before and after the age of menopause (with age-related differences starting to be noticeable at around 45 yr old and disappearing around 55 yr old), while men showed a more linear phenotype association with age (Supplementary Table 2 and Extended Data Figs. 1–3). This pattern remained similar after correction for BMI, smoking and hormone therapy. Adjustment for other major cardiovascular disease (CVD) risk factors affected the significance of five phenotypes with a Cohen’s *f*^2^ close to 0.01: the interaction effects for hematocrit, uric acid and systolic blood pressure (SBP) became nonsignificant, while interaction effects for leukocyte count and C-reactive protein became significant; however, the pattern of age-dependent sex differences remained similar (Supplementary Table 2 and Supplementary Fig. 2). A sliding window *t*-test approach confirmed that the strongest phenotype difference occurs around 50 yr old in women, while age-related differences for men are less significant at any age (Fig. 2a and Supplementary Table 5). Moreover, only 7 of 16 phenotypes showed a significant age by sex interaction when excluding the menopause-related period (45–55 yr old) (Supplementary Table 6). These seven phenotypes comprised five lipid traits, albumin levels and diastolic blood pressure (DBP) and were different between men and women only before menopause. Sex differences were slightly larger in effect size before menopause as compared with after (two-sided Wilcoxon *P* = 0.02).

**Fig. 3:**
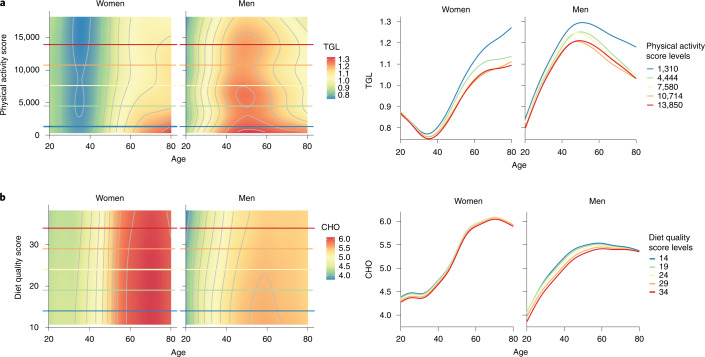
Lifestyle affects phenotypes in an age- and sex-dependent way. **a**,**b**, The age-dependent effect of physical activity score on triglyceride levels (**a**) and of diet quality score on total cholesterol levels (**b**) in women and men, shown by two types of plots. On the left are rasterized heatmap and contour plots where the *y* axis depicts lifestyle factor levels (plotted from the 1st to the 99th percentile), the *x* axis shows age and color reflects phenotype levels. Colored horizontal lines correspond to five values of the lifestyle factor, ranging from the 5th to the 95th percentile. The plots on the right show the relationship of phenotype (*y* axis) with age (*x* axis) by five levels of the lifestyle factor ranging from the 5th to the 95th percentiles. CHO, total cholesterol levels; TGL, triglyceride levels. Source data

The phenotypes showing the strongest menopause effect are related to electrolytes, including plasma levels of calcium, sodium and phosphate (Cohen’s *f*^2^ > 0.02) (but not potassium), supporting previous reports^37,38^ (Fig. 2b and Extended Data Fig. 2). Elevated electrolyte levels have been associated with CVD incidence^39–41^. It has previously been shown that sex hormones and parathyroid hormone affect plasma levels of these electrolytes^42,43^, which likely explains the differences observed around the age of menopause. In line with the known increased prevalence of nonalcoholic fatty liver diseases in postmenopausal women^44^, liver function parameters (alkaline phosphatase, alanine aminotransferase (ALT) and aspartate aminotransferase (AST), but not gamma-glutamyl transferase) also showed a menopause-related age effect (Fig. 3c and Extended Data Fig. 2). These findings are consistent with the menopause-associated difference in AST levels previously reported by Petroff et al.^45^, and we see a similar pattern for ALT levels in our data. Although we observed no age effect on sex differences in serum creatinine levels, other renal function markers such as serum uric acid and urea concentrations tended to become positively associated with age starting from the age of menopause, in line with previous reports^46^ (Extended Data Fig. 2). Blood cell counts showed divergent patterns. Neutrophils were strongly negatively and erythrocytes positively associated with age in women around the age of menopause, an association that has previously been explained by a decrease in estradiol levels and an increase in neutrophil apoptosis rates^47^ (Extended Data Fig. 1). Monocytes, eosinophils, basophils and thrombocytes did not show substantial age by sex interaction.

Menopause-associated phenotype changes are generally thought to be caused by hormonal changes. Using available information about drug intake, we found that for six of ten menopause-associated phenotypes, hormonal medications could delay the onset of the strong phenotype difference by approximately 5 yr (Supplementary Fig. 3). However, these results may be confounded by the low number of postmenopausal women taking hormonal medication (320 women older than 55 received hormonal medication).

### Age- and sex-dependent effects of lifestyle factors

**Extended Data Fig. 4:**
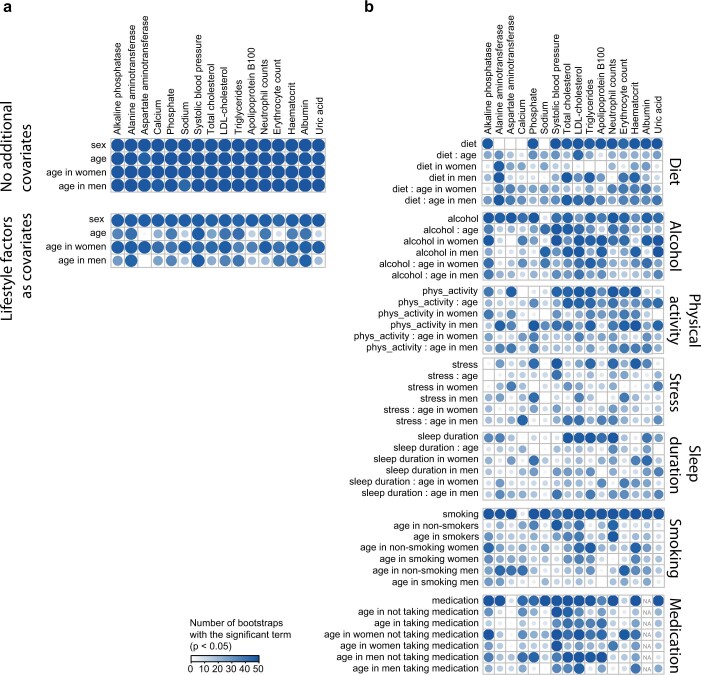
Lifestyle factors affect blood phenotypes in an age- and sex-dependent way. phenotypes showing a significant age by sex interaction (columns) were fitted using GAMs with age, sex, lifestyle factors and all their 2- and 3-way interactions as predictors (rows). 50 bootstraps were run to evaluate model stability. The circle size and color reflect the number of bootstraps in which the corresponding predictor term was significant, that is predictors with a dark blue circle are important for predicting the phenotype and predictors with a white/light blue circle have no or a weak effect on the phenotype. (A) Lifestyle factors partly explain age-dependent sex differences in phenotype levels. The plots reflect the comparison of term significance without (upper plot) and with (bottom plot) lifestyle factors as covariates. (B) Lifestyle factors affect blood phenotypes in an age- and sex-dependent way. No medication was used for albumin levels, therefore the corresponding cells contain NAs. Source data

We checked if any of the observed patterns of significant age by sex interactions were driven by lifestyle factors (current smoking, chronic stress score, physical activity score, diet quality score, alcohol consumption, average sleep duration and medication usage). While we saw that age by sex interactions remained significant for each of the phenotypes, the effect of age for AST was significant only in women after correcting for lifestyle factors. A similar pattern (although with less confidence) was observed for sodium, uric acid and apolipoprotein B100 (Extended Data Fig. 4a).

Next, we studied the differences across age groups in the sex-differential effects of lifestyle on phenotypes. All of our lifestyle factors showed significant interactions with age, sex or both for at least one phenotype (Extended Data Fig. 4b). We observed that alcohol affects all of the tested phenotypes, and diet affects most of them, with the exceptions of AST, sodium and calcium (Extended Data Fig. 4b). The effect of diet quality on total cholesterol was stronger in men than in women, in line with previous reports (Fig. 3)^48^. We saw that lower physical activity is associated with higher triglyceride levels in both sexes, in line with previous studies^49^, and this effect increases with age, which has not been reported before.

### Age-dependent sex differences in cardiometabolic risk factors

Because CMDs exhibit strong age and sex differences, we used our results to characterize age-related sex differences in phenotypic traits relevant for CMD. Among all Lifelines individuals, there were 3,687 patients with CVD (67% males and 33% females), including patients with stroke, heart attack or those who underwent balloon angioplasty or bypass surgery. CVDs occurred more frequently in men than in women of the same age, in line with previous studies (Supplementary Fig. 4)^12^.

**Fig. 4:**
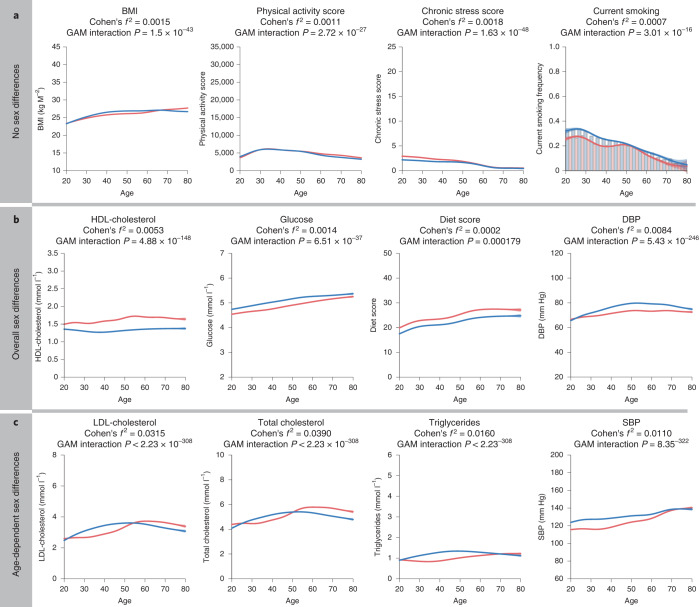
Age-related changes in CMD risk factors in men and women. **a**, CMD risk factors that show a small effect size for sex differences estimated over the whole age span and age-dependent sex differences (Cohen’s *f*^2^ < 0.01). **b**, CMD risk factors that showed sex differences estimated over the whole age span (Cohen’s *f*^2^ ≥ 0.01) and no age-dependent sex differences (Cohen’s *f*^2^ < 0.01). **c**, CMD risk factors that showed age-dependent sex differences (Cohen’s *f*^2^ < 0.01). Lines correspond to the fitted GAMs: blue line represents men, red line represents women. Confidence intervals (±1.96 s.e.m.) are plotted around the lines in a more transparent hue. Cohen’s *f*^2^ reflects the effect size of the age by sex interaction. GAM interaction *P* represents the significance of the age by sex interaction term. Source data

Next, we zoomed in on established CMD risk factors. We investigated the factors that are considered to have a causal effect on CMD, such as blood pressure^24^ or LDL cholesterol levels^23^, as well as risk factors for which there is less evidence for their causality in CMD such as HDL cholesterol or diet quality. Most of these risk factors did not show significant age-related sex differences (Cohen’s *f*^2^ < 0.01), for instance, BMI levels were similar in both sexes (Fig. 4a), in line with previous reports^50^. Several risk factors exhibit strong sex differences that do not differ with age: fasting glucose levels are higher in men than in women, in line with previous reports^51^, whereas HDL cholesterol is higher in women than in men^13^ (Fig. 4b). SBP shows a significant age by sex interaction: while men have higher SBP than women at younger ages, the effect of age on SBP is stronger in women than in men (Cohen’s *f*^2^ = 0.01). DBP is similar in both sexes before age 25 yr and then shows a stronger association with age in men compared with women until age 50, in line with previous reports^52^; however, this age effect is weak (Cohen’s *f*^2^ = 0.008).

In contrast, we see especially strong age-related sex differences in lipid profiles (Fig. 4c). While middle-aged men have higher levels of total cholesterol and LDL cholesterol compared with women, this changes after the age of 50, when women have higher lipid levels than men. This pattern has been reported before^13–16^ and is in line with an increase in CVD risk in postmenopausal women^53^. However, interestingly, the age-related differences in women’s lipid levels are much weaker in effect size and can be observed earlier than many of the other clinical blood phenotypes: the strongest age effect in women occurs between 35 and 55 yr of age. Triglyceride levels in men follow a similar trajectory to total and LDL cholesterol, whereas the age-related patterns between these lipids are different in women. We specifically checked whether these patterns are confounded by use of anti-hypertensive (Supplementary Fig. 5) or lipid-lowering medication (Supplementary Fig. 6), or sex hormone therapy (Supplementary Fig. 3). While use of these medications changes the absolute levels of CMD-related parameters, the age-dependent pattern of sex differences is similar in medication users and nonusers (Supplementary Figs. 3, 5 and 6).

As CMDs are known to be strongly affected by lifestyle^54^, we studied age-related sex differences in smoking, alcohol consumption, physical activity, diet and average sleep duration. In our data, women generally tended to have a better lifestyle at all ages. Diet, for instance, was considered to generally be of better quality in women at all ages, in line with previous studies^55^. In line with national statistics, women smoke less and drink less alcohol than men^56,57^. The physical activity score^58^ was similar in both sexes (Fig. 3a). It is known that women report more stress than men^59^, and our data suggest that this difference is smaller at older age, although the age by sex interaction effect is low (Cohen’s *f*^2^ = 0.002) (Extended Data Fig. 3).

Lifelines participants were followed up for 10 yr, and we used the longitudinal data to estimate the effect of the phenotypes measured at baseline on new-onset CVD events. Overall, 1,436 new CVD cases occurred in both sexes (55% males and 45% females) in the 77,348 participants who were healthy at baseline (Supplementary Fig. 7). We found that 27 of the 51 studied phenotypes are significantly associated with the new CVD events (Supplementary Table 7). For nine of them (33%), the association with CVD was also dependent on age or sex. For example, the effect of DBP on CVD occurrence depended on age and the association of physical activity score with CVD risk was different in men and women (Supplementary Table 7).

#### Pronounced age-dependent sex differences in blood lipid levels

**Fig. 5:**
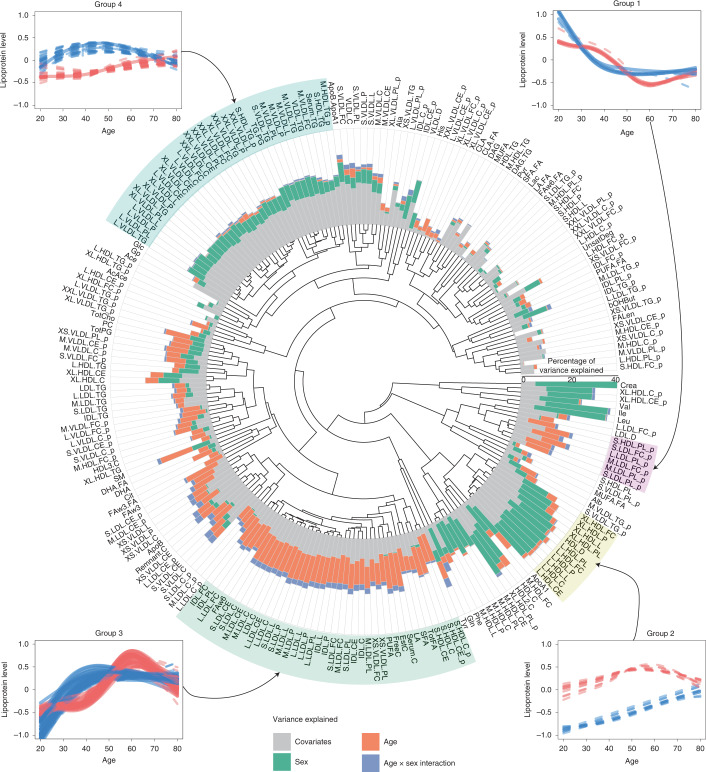
Major patterns of age-dependent sex differences in lipoproteins. For each lipoprotein trait, the fraction of total variance explained by sex (green), age (orange) and age by sex interaction (blue) in addition to covariates (gray) is visualized in the circular bar plot. Bar height reflects the proportion of variance explained. The inner part of the plot is a dendrogram reflecting clustering of age-dependent patterns of sex differences. To illustrate these patterns, four cluster groups were selected. For each group (highlighted in color), plots of the trajectories of its lipoprotein members are shown in the corners for men (blue) and women (red), based on the fitted GAMs. Scaled levels of lipoproteins are depicted on the *y* axis. Solid lines denote lipoproteins with a significant age by sex interaction (*P*_adj_ < 0.05 and Cohen’s *f*^2^ ≥ 0.01). Dotted lines denote lipoproteins for which the age by sex interaction is not significant. Abbreviations of lipoprotein names are expanded in Supplementary Table 8. Source data

To further characterize the strong effect of age-related sex differences in cholesterol and triglyceride levels, we investigated these patterns using 231 metabolic traits (mainly lipid and lipoprotein parameters) measured with a nuclear magnetic resonance spectroscopy (NMR) platform (Nightingale) in a subset of 1,440 participants (Supplementary Fig. 8 and Supplementary Table 8)^60^. More than half of the traits (155 traits, 67%) exhibit an overall nonlinear sex difference, while strong age by sex interaction effects were observed for 73 traits (32%). For each metabolic trait, we calculated the impact of age, sex and their interaction on overall trait variance (Supplementary Table 9 and Fig. 5). The metabolites showing the strongest effect of sex were creatinine, leucine and isoleucine (34%, 30% and 26% of total variance explained, respectively). The effect of age was weaker than that of sex, with the top metabolic parameters affected by age being omega-3 fatty acids, total cholesterol and sphingomyelins (15%, 14% and 14% of variance explained, respectively). Even though age by sex interaction explains less variation than age and sex, it contributes to >3% of variance for some metabolites such as glutamine, tyrosine and remnant cholesterol (Fig. 5).

As many lipid-related traits measured by NMR are correlated, we clustered the observed patterns of age-related sex differences and visualized some examples of distinct groups. Group 1 mainly includes LDL-related parameters, in which the phospholipid/total lipids ratio appears to play the most important role. This group is predominantly affected by age (explaining 10% of its variance, as estimated by permutational multivariate analysis of variance (PERMANOVA); Supplementary Table 10) and exhibits significant age by sex interaction (five of seven metabolic parameters have a significant interaction effect; 2% of variance explained by the interaction term). Group 2 includes large and extra-large HDL parameters, with a strong effect of sex, which explains 21% of variance. This sex difference decreases with age, but the age by sex interaction is not significant. Group 3 primarily includes LDL composition parameters, which are affected by age, which explains 12% of the variance. This group shows a distinct aging pattern that is different in men and women: the highest level in men occurs at 45 yr old, whereas in women these lipoproteins become positively associated with age after 40 yr of age, reach a maximum that surpasses levels in men by age 60 and then become negatively associated with age again, with levels in men and women becoming similar by age 80. Group 4 (8% of variance explained by sex) mostly consists of very-low-density lipoprotein-related parameters that show a nonlinear positive association with age in men until around 50 yr of age, when they become negatively associated with age, while women show a mostly linear association with age (Fig. 5 and Supplementary Tables 9 and 10).

We compared our results with those from a recent study with measurements of metabolites at four timepoints, two of which (25 and 50 yr old) fall into our studied age interval. The concordance in the direction of the sex effect is limited (63% of metabolites show the same effect direction at 25 and 52% at 50), which can be explained by differences in methodology, sample sizes and cohort effect. The higher discordance in our results at 50 yr old confirms our observation that this age is when phenotype levels in men and women become more similar.

Notably, 33 of the 231 NMR lipoproteins were previously found to be able to predict CVD incidence in 10–15 yr, independent of other established risk factors^61^. In our data, 11 of them showed a significant age by sex interaction (Supplementary Table 8). For example, the very-low-density lipoprotein particle concentration, which increases the risk of CVD, shows a positive association with age in men until age 45 and a negative association with age thereafter, but a positive linear association with age in women. Phenylalanine, one of the few NMR-profiled amino acids, is higher in men than women at all ages. Overall, all 33 traits exhibited some level of sex differences that reached nominal significance (*P* < 0.05) in either GAM or linear modeling analyses.

In general, the metabolomics data show that sex differences in most lipoproteins are age-dependent, with remarkable differences in established CVD-associated traits. These CVD-associated particles show divergent patterns of age-related sex differences. In most of the patterns, age starts to affect lipoprotein levels earlier in men than in women; however, the age effect is stronger in women and thus after 50–60 yr old lipoprotein levels in women become more extreme than in men.

#### Age-dependent sex differences in CVD-related proteins

**Extended Data Fig. 5:**
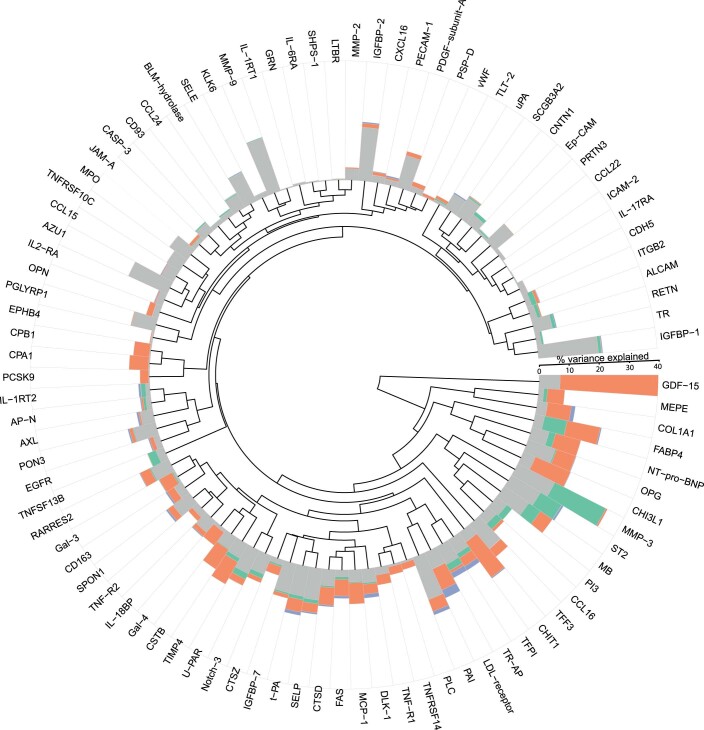
Variance in protein levels explained by age, sex and their interaction. The fraction of total variance explained by sex (green), age (orange) and age by sex interaction (purple) in addition to covariates (grey) is visualized in the circular bar plot for each protein. Bar height reflects the explained variance proportion. The inner part of the plot represents hierarchical clustering of age-dependent patterns of sex differences. Source data

**Extended Data Fig. 6:**
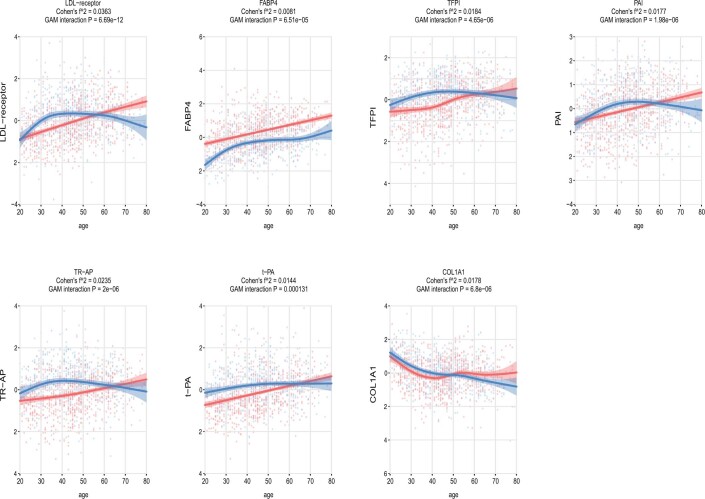
Age-dependent sex differences for CVD-related circulating proteins. For each protein, the plots show scaled values for each person and fitted lines obtained using GAMs: men (blue) and women (red). Confidence intervals (+/− 1.96 SE) around the lines are depicted in a more transparent hue. Fsq (Cohen’s *f* ^2^) reflects the effect size of the age by sex interaction. GAM interaction p indicates the significance of the age by sex interaction term. Only results with a significant age by sex interaction after Bonferroni correction are plotted. Source data

It is often suggested that serum proteomics should be used in addition to major CMD biomarkers and risk factors in disease diagnostics. We therefore aimed to explore how sex differences in proteomics differ with age. For the LifeLines-DEEP (LLD) cohort, a subset of 1,447 samples, we profiled serum proteins that represent established or potential CMD biomarkers using the Olink CVDIII assay. We observed that the effect of age on protein levels is stronger than that of sex. GDF-15 is the protein most affected by age, with 32% of its variance explained, and MMP-3 is the one most affected by sex, which explains 19% of its variance (Extended Data Fig. 5 and Supplementary Table 11). Age-related sex differences in these proteins are less pronounced than in the clinical phenotypes discussed above, with only 7 of 92 proteins showing a significant age by sex interaction after Bonferroni adjustment (Supplementary Table 12 and Extended Data Fig. 6). The seven proteins showing signatures of age by sex interaction include TFPI, PAI, TR-AP and COL1A1, which are considered potential biomarkers of CVD^62–64^. To our knowledge, this is the first observation of an age–sex interaction effect for PAI and TR-AP.

## Discussion

In this work we present a systematic investigation of the age effect on sex differences for 51 clinical phenotypes, with a focus on cardiometabolic risk factors. We performed this analysis in a large population cohort of 146,021 participants aged 20 to 80 yr, which allowed us to estimate the age effect on sex differences with high resolution. In general, blood clinical parameters showed a strong age effect in women around the age of menopause. CMD risk factors showed all varieties of age-related sex differences, ranging from no sex differences (for example, for BMI or physical activity score) to complex patterns of age and sex difference (for example, for blood pressure or lipid levels).

It is well known that sex differences in clinical phenotypes are widespread. While many studies on this topic have been published, many do not take age into account and use it as a covariate or group individuals into age bins^6,65^. This does not, however, allow for identification of the age associated with phenotype changes. Here we show that studying sex differences without taking age into account can lead to misleading conclusions. We find that phenotype differences between men and women are not static, and we observed age by sex interaction for 31% of the clinical traits we investigated. Some parameters, such as total cholesterol levels or neutrophil counts, do not show a sex difference when estimated over the whole age range but are significantly different between men and women when estimated separately before and after age 50. These results are yet another reminder that the reference intervals used in laboratory tests should take both age and sex into account.

We found that for 62% of all parameters with an age by sex interaction effect that are measured in a standard blood test, the effect of age on the phenotype levels strongly increases in women around the age of menopause. These include electrolyte levels and liver and kidney function parameters. In contrast, these phenotypes show a moderate almost linear effect of age in men. When we excluded the menopause period, only seven phenotypes showed age by sex interaction, all before menopause. These seven phenotypes were five lipid traits, albumin levels and DBP, suggesting that the mechanisms underlying these phenotypes are potentially less dependent on menopause. After menopause, no age effect on sex differences in phenotype levels was observed. Menopause and the associated hormonal changes have a profound effect on the total body homeostasis. While the exact mechanism behind this change is not completely understood, it is thought that the decline in estrogen plays a major role in these alterations^66^. One of the strongest changes that we detect at the age of menopause is for blood electrolyte levels, most likely as a consequence of estrogen’s effect on parathyroid hormone levels, kidney function and body fluid regulation^42,67^. We examined the role of estrogen in driving menopause-associated changes by profiling age-related phenotype changes in women taking estrogen medications. Indeed, in these women the effect of age is weaker at time of menopause. Multiple phenotypes nevertheless change slowly in these women and have similar levels with those in women not taking estrogen by the age of 70. A strong menopause-associated age effect in blood parameters is often associated with an increase in disease risk. In our study, liver function markers (AST, ALT and gamma-glutamyl transferase) are strongly positively associated with age around the age of menopause, most likely for several reasons, including an increase in liver disease risk after menopause^44^ and medication usage. These findings highlight the importance of careful health monitoring of women during menopause.

Next, we aimed to characterize the patterns of age-related sex differences of cardiometabolic risk factors that were profiled among the 51 phenotypes studied. Our results support the known age and sex difference for a large fraction of CMD risk factors. In addition, we observe that some of these differences between men and women are also dependent on age, with the largest age effect observed in lipid levels and SBP. We see particularly strong age-related sex differences for lipid levels, which is in line with previous studies^13,16^. Total cholesterol, LDL cholesterol and triglyceride levels are higher in younger men than in women, but women reach similar, or even higher, levels compared with men by the age of 55–60 yr. This increase in the age effect on lipid levels in women starts before menopause, at approximately age 40, and only becomes stronger at the age of menopause, suggesting alternative mechanisms in addition to hormone changes, in line with recent findings that age at natural menopause is not associated with CVD incidence^68^. These lipids are considered to have a causal effect on CVD^21–23^; thus, these findings suggest that disease prevention strategies may need to start at different ages in men and in women.

Further NMR profiling of 231 lipoproteins in a subset of 1,440 individuals allowed us to classify lipoproteins into groups based on the age and sex contribution to variance in their levels and on patterns of age-dependent sex differences. A previous study on age-related lipid trajectories reported three groups as it focused on LDL cholesterol, HDL cholesterol and triglycerides but did not take sex into account^16^. In addition to replicating these published clusters, we see more divergent patterns of age-dependent sex differences, including lipids known to be associated with CVD^61^, highlighting how differently CVD biomarkers can behave with respect to age-dependent sex differences.

Lifestyle factors are known to have a strong effect on human phenotypes, which is particularly important as they can be modified to improve health. We therefore aimed to identify lifestyle factors such as diet quality that explain the observed age-dependent sex differences. However, we found that most of these age-dependent sex differences are not driven by lifestyle factors. In addition, we found profound sex and age differences modifying the effect of lifestyle factors on phenotypes, suggesting the importance of a sex- and age-tailored approach in disease prevention based on lifestyle modification.

**Extended Data Fig. 7:**
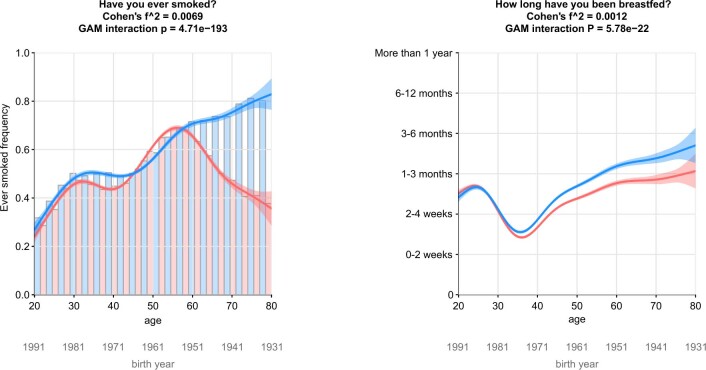
The effect of generation confounds age differences. Sex differences in the fraction of individuals who have ever smoked (left) and the duration of breastfeeding (right) are dependent on external factors related to specific generations rather than actual age. The plots show fitted lines obtained using GAMs: men (blue) and women (red). Confidence intervals (+/− 1.96 SE) around the lines are depicted in a more transparent hue. Source data

We acknowledge several limitations of our study. The main limitations of our study are the self-reported CMD status, the limited age range (20–80 yr) and the cross-sectional design. The fact that most of our phenotype data are cross-sectional does not allow us to distinguish a genuine age effect from a generation-associated effect. For example, our data show that older people are shorter in height, which reflects a well-studied increase in height in the Netherlands in the 20th century, which is related to environmental causes and natural selection^69^ rather than the effect of age. We also observed generation effects for other phenotypes not explicitly discussed in this work. Our results suggest that in the 20th century duration of breastfeeding decreased and was the lowest in the 1970s, probably due to the increasing availability of commercially prepared formulas^70^ (Extended Data Fig. 7). In addition, it seems that before the 1980s boys were breastfed longer than girls. We also see a generational effect in the age by sex interaction pattern of the phenotype reflecting if a person has ever smoked. Women born before 1950 smoked less often than men, while women born in the 1950s to 1960s are of the generation where women began to smoke often, sometimes even more than men (Extended Data Fig. 7). Large longitudinal datasets will be needed to exclude such generational effects.

Over the last decades, an impressive effort has been made to improve CVD prevention, diagnosis and treatment in women. However, much can be done to create sex-tailored prevention and treatment strategies, the efficacy of which largely depends on information about risk factors. The risk factors that have the strongest prognostic power for CVD are age, sex and ethnicity, which are all nonmodifiable factors^71^ that act by affecting a wide range of other phenotypes and as a consequence of the disease itself. Thus, it is extremely important to study the effect of age and sex on other CVD risk factors to be able to improve the way we monitor risk factor levels and prevent disease. We believe that our results will help to improve understanding of the relationship between age, sex and CVD risk factors. Future large longitudinal studies (including follow-up of this cohort) are crucial to link the age dynamics of sex differences in these risk factors to the long-term effect on diseases and to provide options for early disease prevention.

## Methods

### Data description

Lifelines is a multidisciplinary prospective population-based cohort study using a unique three-generation design to examine the health and health-related behaviors of 167,729 individuals living in the northeastern region of the Netherlands. It employs a broad range of investigative procedures to assess the biomedical, socio-demographic, behavioral, physical and psychological factors that contribute to the health and disease of the general population, with a special focus on multi-morbidity and complex genetics^34,72^. The Lifelines study was approved by the ethics committee of the University Medical Center Groningen, document number METc2007/152. All participants signed an informed consent form before enrollment.

In this study, we used the phenotype data collected from all available individuals at the first visit. We kept only samples for which there was sex and age information and fasting blood test measurements and focused on the age range from 20 to 80 yr due to low sample size outside this range. This resulted in data for 146,021 individuals (58% female). For these samples, we examined 51 phenotypes representing 35 blood test parameters, 4 blood pressure measurements, 6 anthropometric measurements, 6 lifestyle factors (current smoking, diet score^73^, Short QUestionnaire to ASsess Health enhancing physical activity (SQUASH) total physical activity score^58^, alcohol consumption, chronic stress score and average sleep duration) and self-reported type 2 diabetes status (Supplementary Table 1). CVD was defined as self-reported stroke, heart attack or angioplasty/bypass surgery.

In addition, we examined a subset of the Lifelines dataset, the LLD cohort, which consists of around 1,500 samples for which several omics layers were profiled^74^. For these samples, we looked at two relevant data types. First, we used plasma levels of 92 proteins measured using the Olink CVDIII panel for 1,447 samples (57% female). These proteins were selected because they are known or potential CVD risk factors and biomarkers. Generation of these data was described previously^75^. Second, we used the NMR lipidomics data for 231 lipid particles that were available for 1,440 samples (57% female), which was also previously described^60^.

### Statistical analyses

Before all analyses, some right-skewed clinical phenotypes were log-transformed (see Supplementary Table 1 for the list of log-transformed phenotypes). Second, extreme outliers were removed by excluding observations that were more than three interquartile ranges below the first quartile or more than three interquartile ranges above the third quartile from the whole dataset, not taking age and sex into account.

We used two approaches to study sex differences. General sex differences present over the whole age span were determined using ordinary least squares linear modeling in R, adjusting for relevant covariates (see Covariate adjustment for description of the phenotypes used). Age-dependent nonlinear sex differences were studied by fitting a GAM with integrated smoothness estimation using the mgcv v.1.8-31 package in R^76^. For all phenotypes, we used spline smoothing, and smoothness selection was done using restricted maximum likelihood. Gaussian family was used for all phenotypes except binary ones such as smoking status, where Binomial family with the logit link function was used. We fitted a model of the following form:

${{{\mathrm{Phenotype\sim GAM}}}}\left( {{{{\mathrm{sex + s}}}}\left( {{{{\mathrm{age}}}}} \right){{{\mathrm{ + s}}}}\left( {{{{\mathrm{age,by = sex}}}}} \right){{{\mathrm{ + covariates}}}}} \right){{{\mathrm{,}}}}$where s(age) denotes a spline smooth of age and s(age, by = sex) is an interaction term with smoothing for age by (ordered) sex interaction. The covariates used for different phenotypes are discussed below. Multiple testing correction was done separately for clinical phenotypes, lipoprotein levels and protein levels using Bonferroni correction. The age of phenotype change was first estimated visually based on the fitted model trajectories, then confirmed using a sliding window *t*-test in which a *t*-test was performed for each year of age, comparing mean phenotype levels in a window of 5 yr before versus 5 yr after that age.

Due to the large sample size, even minor age by sex interaction effects were significant. To select the phenotypes with a considerable age by sex interaction, we estimated the phenotype’s effect size using Cohen’s *f*^2^ (refs. ^77,78^) and a threshold of *f*^2^ ≥ 0.01. Cohen’s *f*^2^ was calculated using the following formula:

$$f^2 = \frac{{R_{\mathrm{full}}^2 - R_{\mathrm{sub}}^2}}{{1 - R_{\mathrm{full}}^2}}$$

where $R_{\mathrm{full}}^2$ is the proportion of variance explained by the model with an age × sex interaction term and $R_{\mathrm{sub}}^2$ is the proportion of variance explained by the model without an age × sex interaction term. In a similar fashion, we estimated the effect size of the overall sex differences when not taking age into account. Overall, we considered phenotypes to show an age by sex interaction if the *P* value of the age × sex interaction term in the GAM was significant after Bonferroni correction for multiple testing and the effect size of this interaction had a Cohen’s *f*^2^ ≥ 0.01.

Finally, we compared the linear model with and without the age by sex interaction term and a GAM model with and without the age by sex interaction term by calculating mean squared error based on tenfold cross-validation using the gamclass v.0.62.3 R package.

#### Covariate adjustment

All selected phenotypes were analyzed using GAMs, first without any covariate adjustment and then adding current smoking, hormone therapy (estrogens and progestogens) and nonlinear BMI effect to the GAM. Hormone therapy was queried only for women by extracting Anatomical Therapeutic Chemical (ATC) code G03, excluding only androgens (G03B) and plain anti-androgens (G03HA). In addition, we checked if the observed patterns are confounded by other CMD risk factors by adding additional covariates to the base model. Here, we used the following nonlinear covariates: BMI, glucose levels, total cholesterol, HDL cholesterol and SBP; and the following linear covariates: current smoking and type 2 diabetes. We excluded a covariate from the model if it is relevant to the phenotype of interest (used as an independent variable). Thus, we did not correct lipid phenotypes for total and HDL cholesterol, blood pressure phenotypes for SBP, weight parameters for BMI and glycated hemoglobin for glucose levels.

The effects of several medication groups on age-related sex differences were investigated by fitting the GAM model separately for men and women and for medication users versus nonusers. First, we did this for hormone therapy (ATC code G03 excluding only androgens (G03B) and plain anti-androgens (G03HA)). Next, we checked the effect on CVD risk factors of other CVD-related medication categories: statins (ATC code C10AA0) and anti-hypertensive drugs (general anti-hypertensives (ATC code C02), diuretics excluding vasopressin antagonists (C03 excluding C03X), beta-blockers (C07), calcium channel blockers (C08) and agents acting on the renin-angiotensin system (C09)). In addition, we checked the effect of calcium supplementation (ATC code A12A) on serum calcium levels.

#### Leave-out analysis to estimate age by sex interactions independent of menopause

To check the effect of age on sex differences excluding the menopause period, we split our cohort into two groups: before menopause (age 20–45) and after menopause (age 55–80) and ran the primary analysis (GAM with age, sex and age by sex interaction terms) in these two groups separately, calculating the number of phenotypes with a significant age by sex interaction (Bonferroni-adjusted *P* < 0.05 and Cohen’s *f*^2^ ≥ 0.01). To compare the sex effect in these two groups, we performed a two-sided Wilcoxon test on the Cohen’s *f*^2^ of the sex term in the GAM run without age by sex interaction.

#### Estimating the effect of lifestyle factors on phenotypes

We estimated the effect of lifestyle factors on phenotypes showing a significant age by sex interaction. For each phenotype, we fitted a GAM with penalization (select = T) to select the significant predictors using the following seven lifestyle factors as predictors: current smoking, chronic stress score (measuring long-term difficulties experienced during the last year), physical activity score, diet quality score, amount of alcohol consumption, average sleep duration per 24 h and medication usage. For each phenotype, we selected a set of relevant medication categories (erythrocyte count: ATC codes B03XA and G03B; neutrophil counts: N05A, J01 and A11; hematocrit: A10; calcium: A12; sodium: N03, C03, C09 and A02BC; phosphate: A02A, C03 and A10A; alkaline phosphatase: G03 and N03; ALT: N03, J01 and M01A; AST: N03, J01, M01A and N02BE01; lipids: C10 and uric acid: C03A, C03C, M04AA01 and M04AB03). No medication was used for blood albumin levels.

For each phenotype, we built a GAM using age, sex and the seven lifestyle factors together with their two-way interactions with age, their two-way interactions with sex and their three-way interactions with both age and sex as predictors and phenotype levels as outcome. Sex, medication and smoking status were added to the model as (nonordered) factors.

When interaction of a continuous variable with a factor is present in the mgcv GAMs, a separate smooth is estimated for a continuous variable for all observations, together with deviations from this smooth for each factor level. Therefore, for each continuous lifestyle factor, there are six terms in the model: (1) a smooth effect of the lifestyle factor for both sexes combined together, (2) its smooth interaction with age for both sexes, (3) the additional smooth effect of the lifestyle factor in men, (4) the additional smooth effect of the lifestyle factor in women, (5) the additional smooth interaction of lifestyle factor with age for men and (6) the additional smooth interaction of lifestyle factor with age for women.

We considered a predictor to be selected during the penalization approach if it had a *P* < 0.05. To test model stability, we ran 50 bootstraps for this model, and, for each predictor, we plotted how many times it was selected as significant by the GAM penalization approach.

#### New-onset CVD prediction model

We further tested if any of the baseline phenotypes could predict CVD events in the following 10 yr. This test was based on information about new CVD cases in Lifelines participants that was collected approximately 10 yr later (by timepoint 3a). Baseline sample inclusion criteria were: no patients with CVD (no stroke, no heart attack and no angioplasty/bypass surgery) and no statins or anti-hypertensive drug users. New CVD cases were defined as those participants who indicated that any of the CVD phenotypes (heart attack, stroke and CVD in general) had occurred since their baseline visit. We thus included the 77,348 participants who had received the questionnaires and fulfilled the inclusion criteria at baseline. Of these participants, 1,436 developed CVD phenotypes within the subsequent 10 yr. We then built a logistic GAM with penalization (select = T) per phenotype in which we tested the effects of age, sex, current phenotype and four interaction terms: age by sex, phenotype by age, phenotype by sex and a three-way interaction of phenotype, age and sex. We controlled false discovery rate by applying Bonferroni correction.

#### Sex–age interaction analysis of lipidomics data

NMR lipidomics data for 231 lipid and lipoprotein traits were available for 1,440 samples. We *z*-transformed the data before analyses to ensure that the different lipoproteins scaled similarly. We corrected these data for covariates by adding current smoking, statin usage and a nonlinear term for BMI to the GAM model. We performed hierarchical clustering (hclust) of the resulting fitted values (predicted at 300 age points for each sex) using Euclidean distance and the unweighted pair group method with arithmetic mean (UPGMA) agglomeration method.

#### Sex–age interaction analysis of CVD-related proteins

For 1,447 samples, plasma levels of 92 proteins were measured using the Olink CVDIII panel. These data were *z*-transformed before analyses. We corrected data for covariates by adding linear terms for blood cell counts, current smoking and hormone therapy (G03 excluding G03B and G03HA), as these covariates were previously found to affect protein levels^75^. We performed hierarchical clustering (hclust) of the resulting fitted values (predicted at 300 age points for each sex) using Euclidean distance and the UPGMA agglomeration method.

#### Calculation of explained variance

We calculated the fraction of total variance of each metabolic and proteomic trait explained by the covariates sex, age and age by sex interaction. To do so, we fitted four separate GAMs and calculated the additional fraction of variance explained by sex, age and age by sex interaction expressed as the squared Pearson correlation between observed and predicted values. To ensure the stability of results, we used the average explained variance obtained from fivefold cross-validation of the procedure described above repeated ten times. The results are presented in a circular bar plot which was plotted using the R package ‘circlize’ v.0.4.14.

In addition, we estimated the variance explained by age, sex and their interaction for all NMR lipoproteins together and for each lipoprotein cluster using PERMANOVA. PERMANOVA was performed using the adonis2 function from the vegan 2.5-6 R package, using Euclidean distance to estimate sample dissimilarity and 1,000 permutations to calculate term significance.

### Reporting summary

Further information on research design is available in the Nature Research Reporting Summary linked to this article.

### Supplementary information

Supplementary Information Supplementary Figs. 1–8.

Reporting Summary

Supplementary Tables Supplementary Tables 1–12.

### Source data

Source Data Fig. 2 Statistical source data.

Source Data Fig. 3 Statistical source data.

Source Data Fig. 4 Statistical source data.

Source Data Fig. 5 Statistical source data.

Source Data Extended Data Fig. 1 Statistical source data.

Source Data Extended Data Fig. 2 Statistical source data.

Source Data Extended Data Fig. 3 Statistical source data.

Source Data Extended Data Fig. 4 Statistical source data.

Source Data Extended Data Fig. 5 Statistical source data.

Source Data Extended Data Fig. 6 Statistical source data.

Source Data Extended Data Fig. 7 Statistical source data.

## Data Availability

Participant phenotype data are not publicly available to protect participants’ privacy. These data can be requested by sending a scientific proposal to the Lifelines Biobank (https://www.lifelines.nl/researcher/how-to-apply). All data access to the Lifelines population cohort must follow the informed consent regulations of the Medical Ethics Review Board of the University Medical Center Groningen described at http://lifelines.nl/. The proteomics data used in this study are available at the European Genome-Phenome Archive under accession EGAD00001009268. Source data are provided with this paper.

## Code availability

Analysis codes are available via: https://github.com/DashaZhernakova/umcg_scripts/tree/master/age_sex_interactions_paper.
