# Publisher Correction: Age-dependent sex differences in cardiometabolic risk factors

**DOI:** 10.1038/s44161-022-00146-1

**Published:** 2022-09-23

**Authors:** Daria V. Zhernakova, Trishla Sinha, Sergio Andreu-Sánchez, Jelmer R. Prins, Alexander Kurilshikov, Jan-Willem Balder, Serena Sanna, Lifelines Cohort Study, Lude Franke, Jan A. Kuivenhoven, Alexandra Zhernakova, Jingyuan Fu

**Affiliations:** 1 Department of Genetics, University Medical Center Groningen, University of Groningen, Groningen, the Netherlands; 2 Laboratory of Genomic Diversity, Center for Computer Technologies, ITMO University, Saint Petersburg, Russia; 3 Department of Pediatrics, University Medical Center Groningen, University of Groningen, Groningen, the Netherlands; 4 Department of Obstetrics and Gynecology, University Medical Center Groningen, University of Groningen, Groningen, the Netherlands; 5 Division of Heart and Lungs, Department of Cardiology, University Medical Center Utrecht, Utrecht University, Utrecht, the Netherlands; 6 Istituto di Ricerca Genetica e Biomedica (IRGB) del Consiglio Nazionale delle Ricerche (CNR), Monserrato, Italy

**Keywords:** Cardiovascular diseases, Biomarkers, Data integration

Correction to: *Nature Cardiovascular Research* 10.1038/s44161-022-00131-8, published online 12 September 2022.

In the version of this article initially published, Lude Franke, Jan A. Kuivenhoven, Alexandra Zhernakova and Jingyuan Fu were listed in the Lifelines Cohort Study group but were omitted from the main author list, while Jan A. Kuivenhoven was mistakenly included in the study group. The errors have been corrected in the HTML and PDF versions of the article.

