# Supplementary material for: Age-dependent sex differences in cardiometabolic risk factors
Source: Nat Cardiovasc Res. 2022 Sep 12;1(9):844–54. doi: 10.1038/s44161-022-00131-8 (PMC11357998; doi:10.1038/s44161-022-00131-8)
Supplement: Supplementary file 1 — Supplementary Figs. 1–8. [file 44161_2022_131_MOESM1_ESM.pdf]

nature portfolio

https://doi.org/10.1038/s44161-022-00131-8

Supplementary information

Age-dependent sex differences in cardiometabolic risk factors

In the format provided by the
authors and unedited

Supplementary Figures

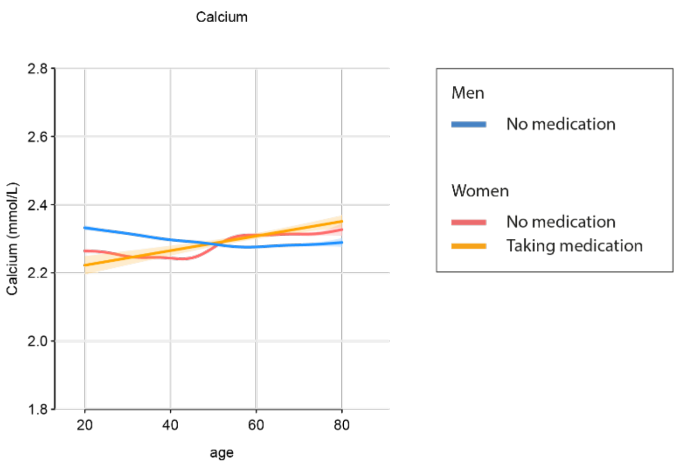

Supplementary Figure 1. The effect of calcium supplementation on serum calcium levels in women.

GAM-based fitted lines are plotted for men (blue) and women (red) not taking calcium supplementation and for women taking calcium supplementation (yellow). Confidence intervals (+/- 1.96 SE) are depicted around the lines in a more transparent hue.

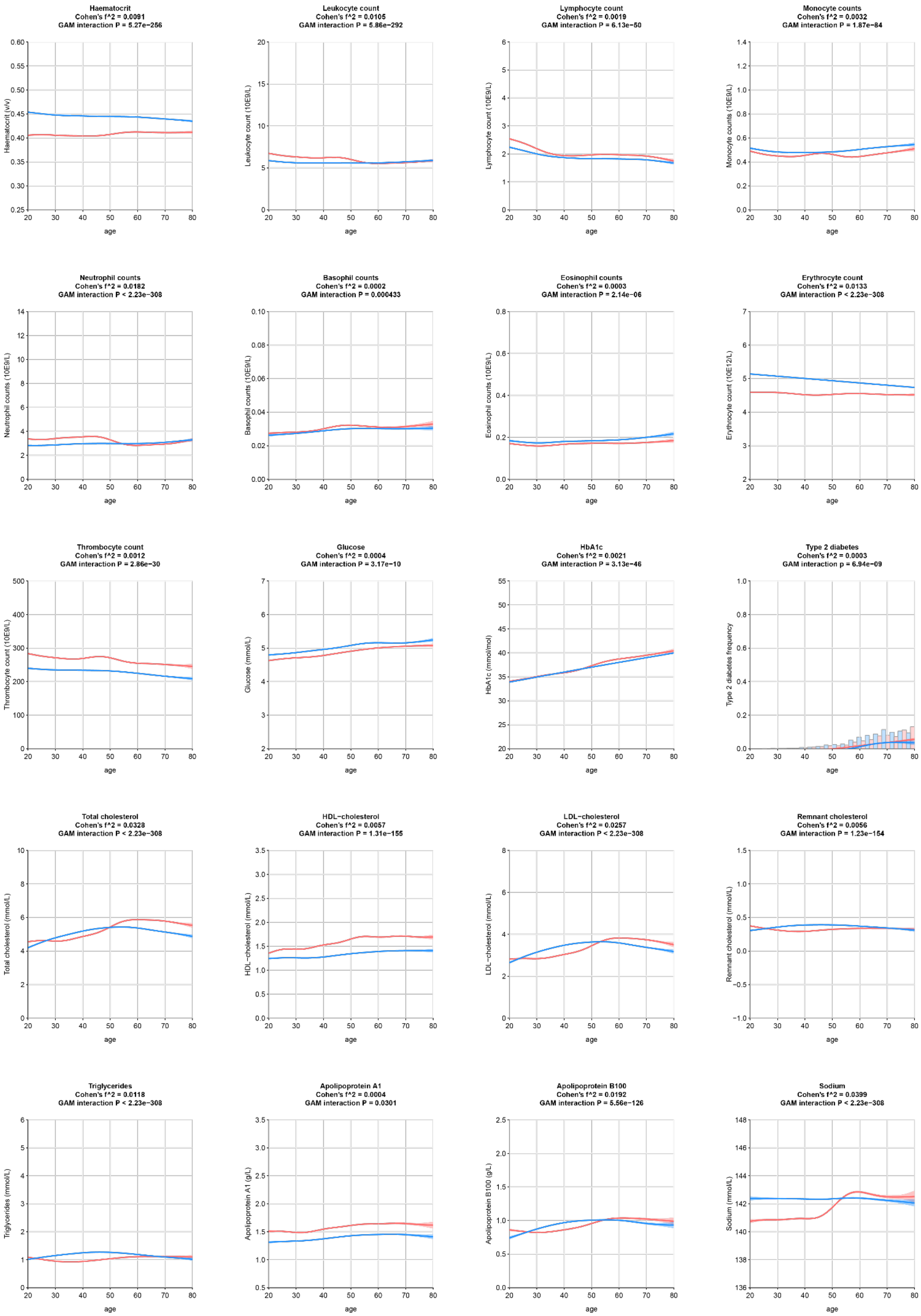
Haematocrit
Cohen's f^2 = 0.0091
GAM interaction P = 5.27e−256
Haematocrit (v/v)
Leukocyte count
Cohen's f^2 = 0.0105
GAM interaction P = 5.86e−292
Leukocyte count (10E9/L)
Lymphocyte count
Cohen's f^2 = 0.0019
GAM interaction P = 6.13e−50
Lymphocyte count (10E9/L)
Monocyte counts
Cohen's f^2 = 0.0032
GAM interaction P = 1.87e−84
Monocyte counts (10E9/L)
Neutrophil counts
Cohen's f^2 = 0.0182
GAM interaction P < 2.23e−308
Neutrophil counts (10E9/L)
Basophil counts
Cohen's f^2 = 0.0002
GAM interaction P = 0.000433
Basophil counts (10E9/L)
Eosinophil counts
Cohen's f^2 = 0.0003
GAM interaction P = 2.14e−06
Eosinophil counts (10E9/L)
Erythrocyte count
Cohen's f^2 = 0.0133
GAM interaction P < 2.23e−308
Erythrocyte count (10E12/L)
Thrombocyte count
Cohen's f^2 = 0.0012
GAM interaction P = 2.86e−30
Thrombocyte count (10E9/L)
Glucose
Cohen's f^2 = 0.0004
GAM interaction P = 3.17e−10
Glucose (mmol/L)
HbA1c
Cohen's f^2 = 0.0021
GAM interaction P = 3.13e−46
HbA1c (mmol/mol)
Type 2 diabetes
Cohen's f^2 = 0.0003
GAM interaction p = 6.94e−09
Type 2 diabetes frequency
Total cholesterol
Cohen's f^2 = 0.0328
GAM interaction P < 2.23e−308
Total cholesterol (mmol/L)
HDL−cholesterol
Cohen's f^2 = 0.0057
GAM interaction P = 1.31e−155
HDL−cholesterol (mmol/L)
LDL−cholesterol
Cohen's f^2 = 0.0257
GAM interaction P < 2.23e−308
LDL−cholesterol (mmol/L)
Remnant cholesterol
Cohen's f^2 = 0.0056
GAM interaction P = 1.23e−154
Remnant cholesterol (mmol/L)
Triglycerides
Cohen's f^2 = 0.0118
GAM interaction P < 2.23e−308
Triglycerides (mmol/L)
Apolipoprotein A1
Cohen's f^2 = 0.0004
GAM interaction P = 0.0301
Apolipoprotein A1 (g/L)
Apolipoprotein B100
Cohen's f^2 = 0.0192
GAM interaction P = 5.56e−126
Apolipoprotein B100 (g/L)
Sodium
Cohen's f^2 = 0.0399
GAM interaction P < 2.23e−308
Sodium (mmol/L)
age

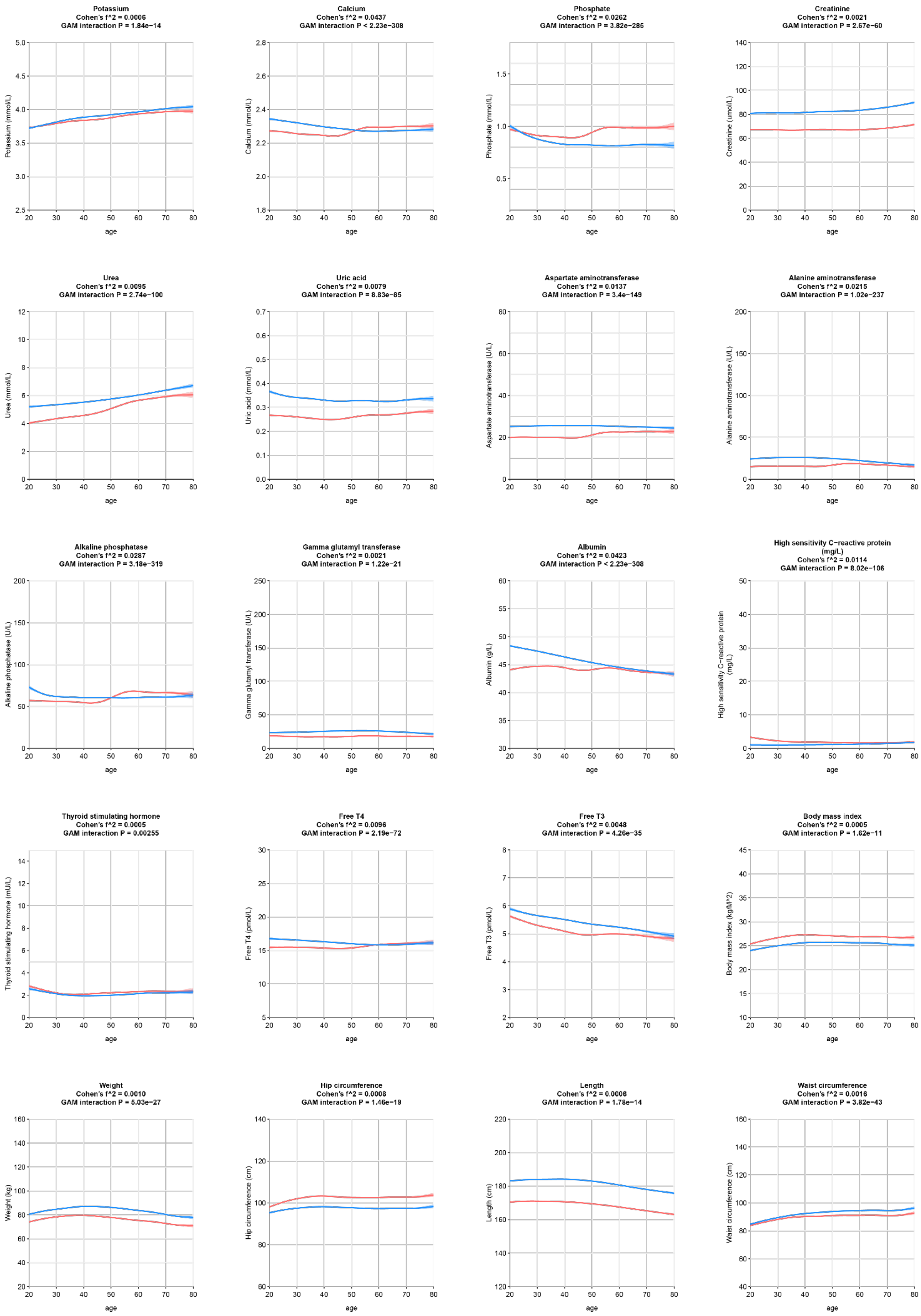
Potassium
Cohen's f^2 = 0.0006
GAM interaction P = 1.84e−14
Potassium (mmol/L)
age
Calcium
Cohen's f^2 = 0.0437
GAM interaction P < 2.23e−308
Calcium (mmol/L)
age
Phosphate
Cohen's f^2 = 0.0262
GAM interaction P = 3.82e−285
Phosphate (mmol/L)
age
Creatinine
Cohen's f^2 = 0.0021
GAM interaction P = 2.67e−60
Creatinine (umol/L)
age
Urea
Cohen's f^2 = 0.0095
GAM interaction P = 2.74e−100
Urea (mmol/L)
age
Uric acid
Cohen's f^2 = 0.0079
GAM interaction P = 8.83e−85
Uric acid (mmol/L)
age
Aspartate aminotransferase
Cohen's f^2 = 0.0137
GAM interaction P = 3.4e−149
Aspartate aminotransferase (U/L)
age
Alanine aminotransferase
Cohen's f^2 = 0.0215
GAM interaction P = 1.02e−237
Alanine aminotransferase (U/L)
age
Alkaline phosphatase
Cohen's f^2 = 0.0287
GAM interaction P = 3.18e−319
Alkaline phosphatase (U/L)
age
Gamma glutamyl transferase
Cohen's f^2 = 0.0021
GAM interaction P = 1.22e−21
Gamma glutamyl transferase (U/L)
age
Albumin
Cohen's f^2 = 0.0423
GAM interaction P < 2.23e−308
Albumin (g/L)
age
High sensitivity C−reactive protein
(mg/L)
Cohen's f^2 = 0.0114
GAM interaction P = 8.02e−106
High sensitivity C−reactive protein
(mg/L)
age
Thyroid stimulating hormone
Cohen's f^2 = 0.0005
GAM interaction P = 0.00255
Thyroid stimulating hormone (mU/L)
age
Free T4
Cohen's f^2 = 0.0096
GAM interaction P = 2.19e−72
Free T4 (pmol/L)
age
Free T3
Cohen's f^2 = 0.0048
GAM interaction P = 4.26e−35
Free T3 (pmol/L)
age
Body mass index
Cohen's f^2 = 0.0005
GAM interaction P = 1.62e−11
Body mass index (kg/M^2)
age
Weight
Cohen's f^2 = 0.0010
GAM interaction P = 5.03e−27
Weight (kg)
age
Hip circumference
Cohen's f^2 = 0.0008
GAM interaction P = 1.46e−19
Hip circumference (cm)
age
Length
Cohen's f^2 = 0.0006
GAM interaction P = 1.78e−14
Length (cm)
age
Waist circumference
Cohen's f^2 = 0.0016
GAM interaction P = 3.82e−43
Waist circumference (cm)
age

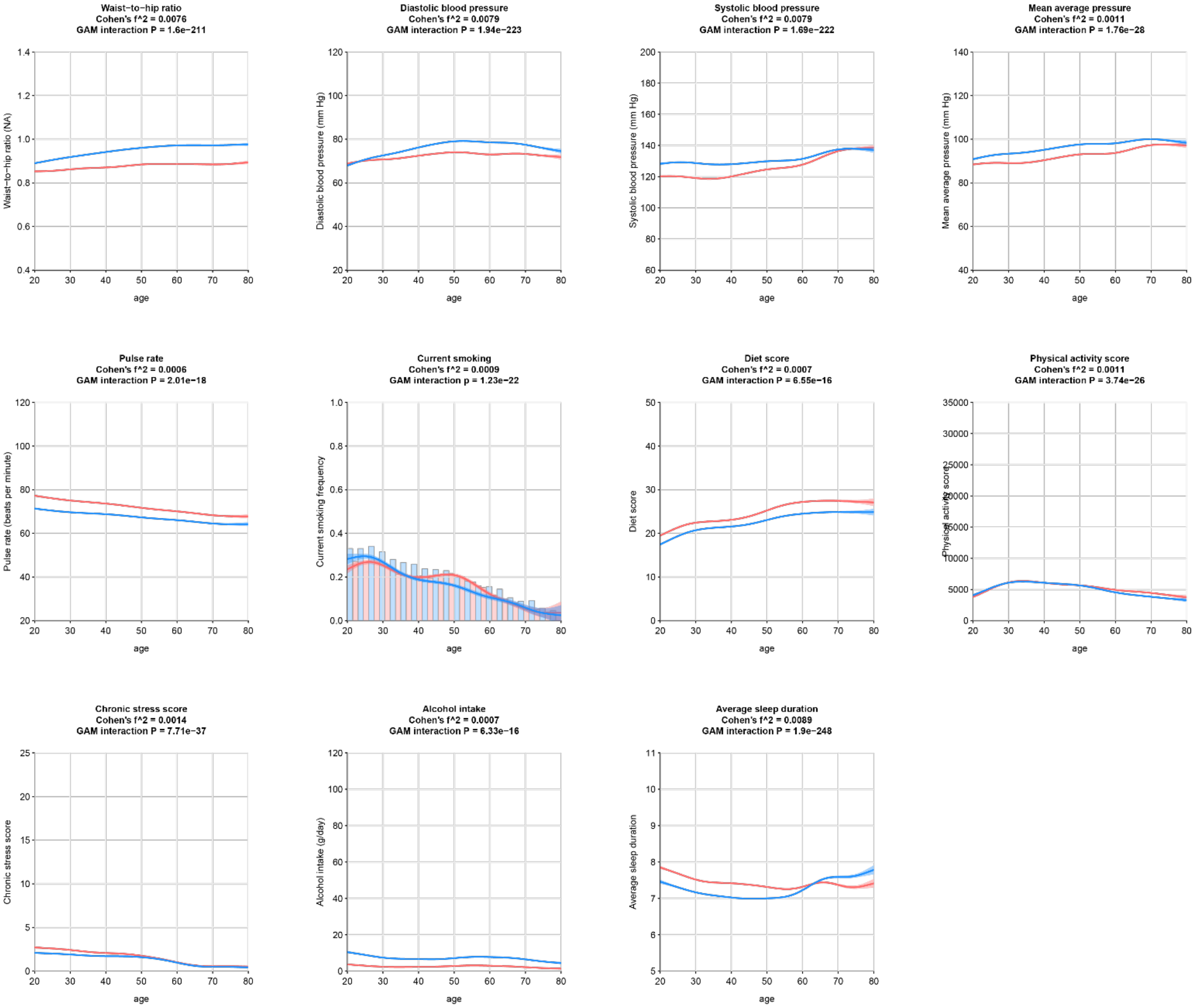

Supplementary Figure 2. Age-dependent sex differences after adjusting for major CVD risk factors

For each phenotype, GAM-based fitted lines are plotted for men (blue) and women (red) after adjusting for the major CVD risk factors: BMI, glucose levels, total cholesterol, HDL-cholesterol, systolic blood pressure, current smoking and type 2 diabetes. Covariates correlated to the phenotype of interest were excluded from the model. Confidence intervals (+/- 1.96 SE) are depicted around the lines in a more transparent hue. Plots are given for all phenotypes without multiple testing correction.

Sex hormone therapy

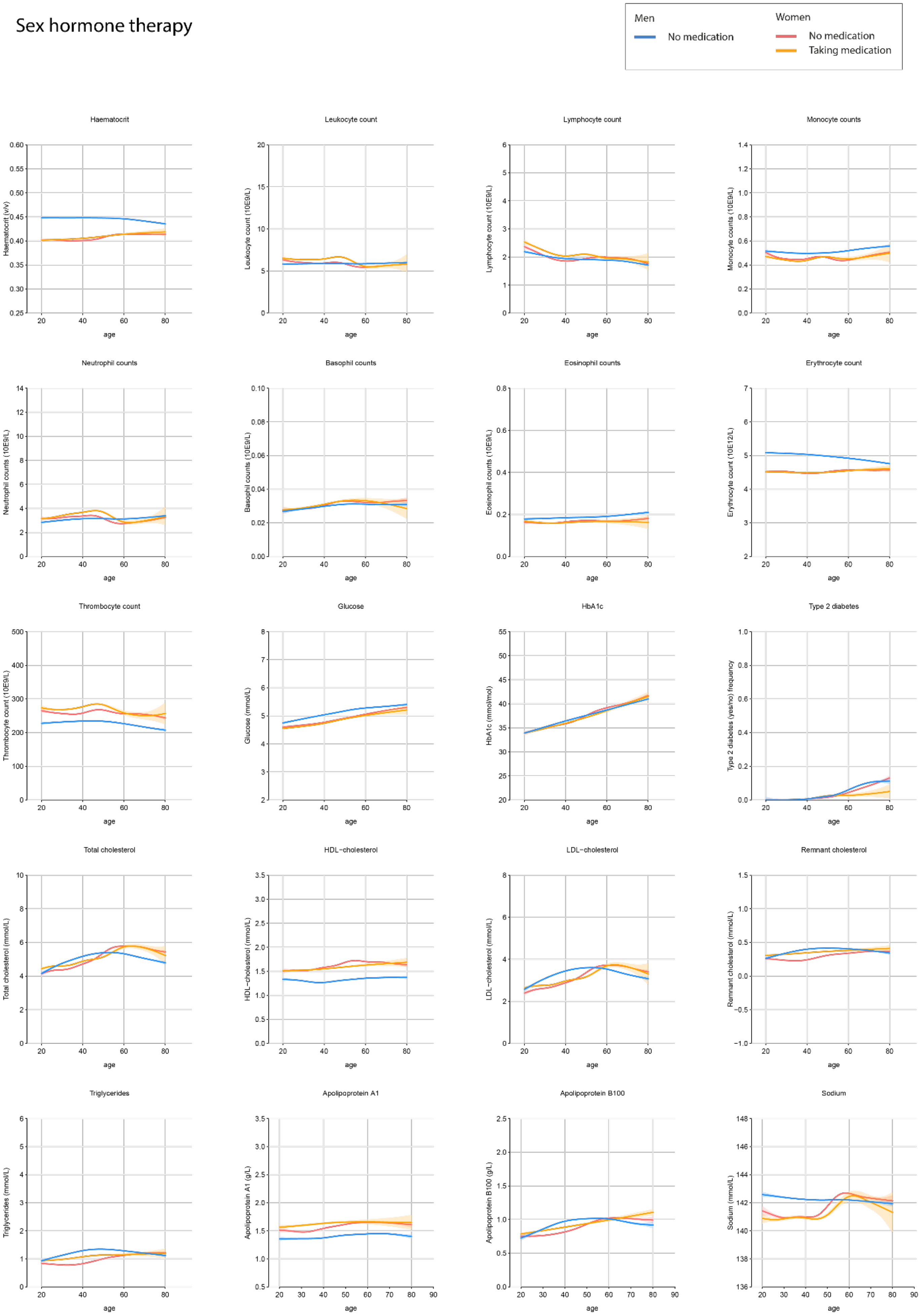

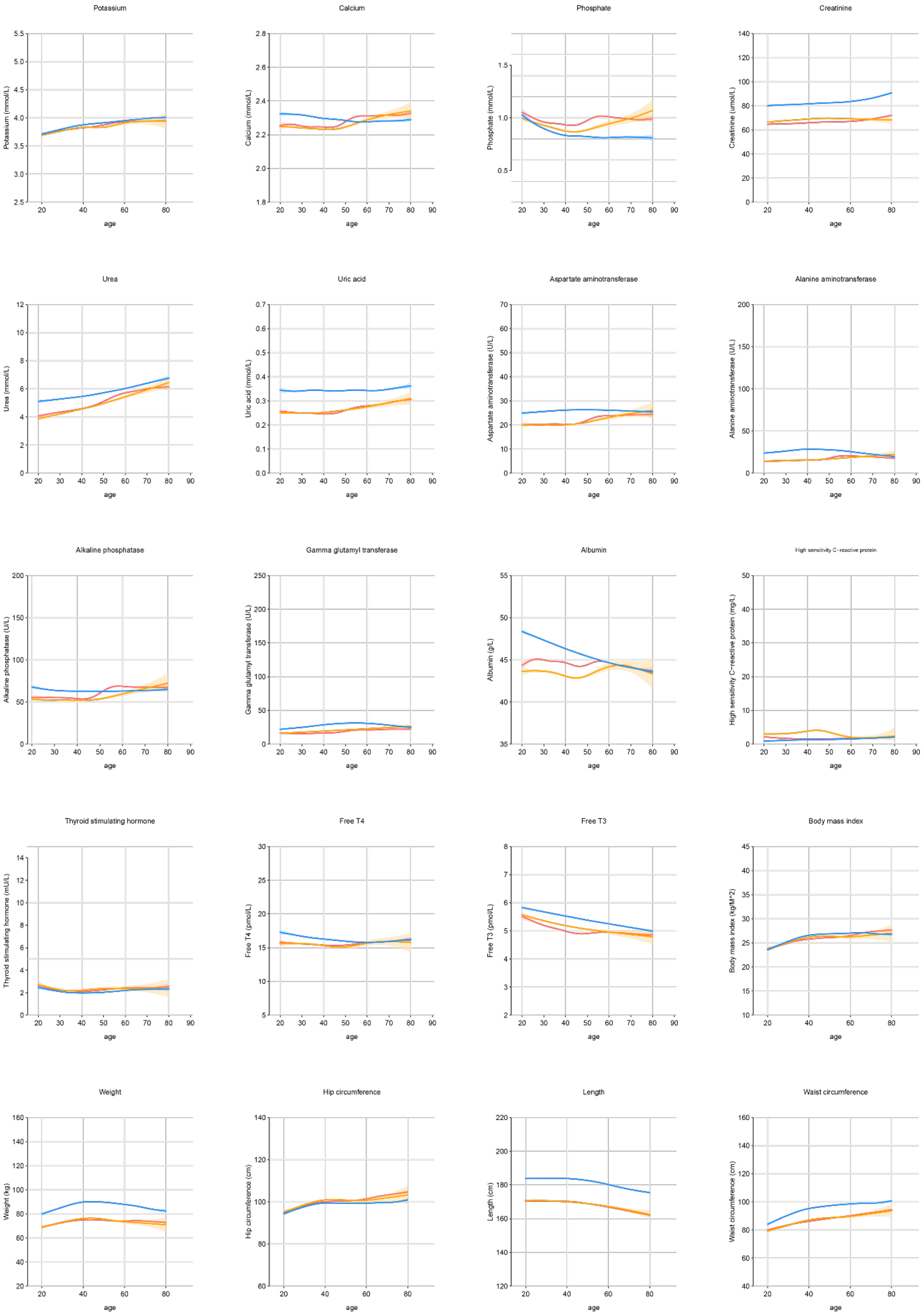
Potassium
Potassium (mmol/L)
Calcium
Calcium (mmol/L)
Phosphate
Phosphate (mmol/L)
Creatinine
Creatinine (umol/L)
Urea
Urea (mmol/L)
Uric acid
Uric acid (mmol/L)
Aspartate aminotransferase
Aspartate aminotransferase (U/L)
Alanine aminotransferase
Alanine aminotransferase (U/L)
Alkaline phosphatase
Alkaline phosphatase (U/L)
Gamma glutamyl transferase
Gamma glutamyl transferase (U/L)
Albumin
Albumin (g/L)
High sensitivity C-reactive protein
High sensitivity C-reactive protein (mg/L)
Thyroid stimulating hormone
Thyroid stimulating hormone (mU/L)
Free T4
Free T4 (pmol/L)
Free T3
Free T3 (pmol/L)
Body mass index
Body mass index (kg/M^2)
Weight
Weight (kg)
Hip circumference
Hip circumference (cm)
Length
Length (cm)
Waist circumference
Waist circumference (cm)
age

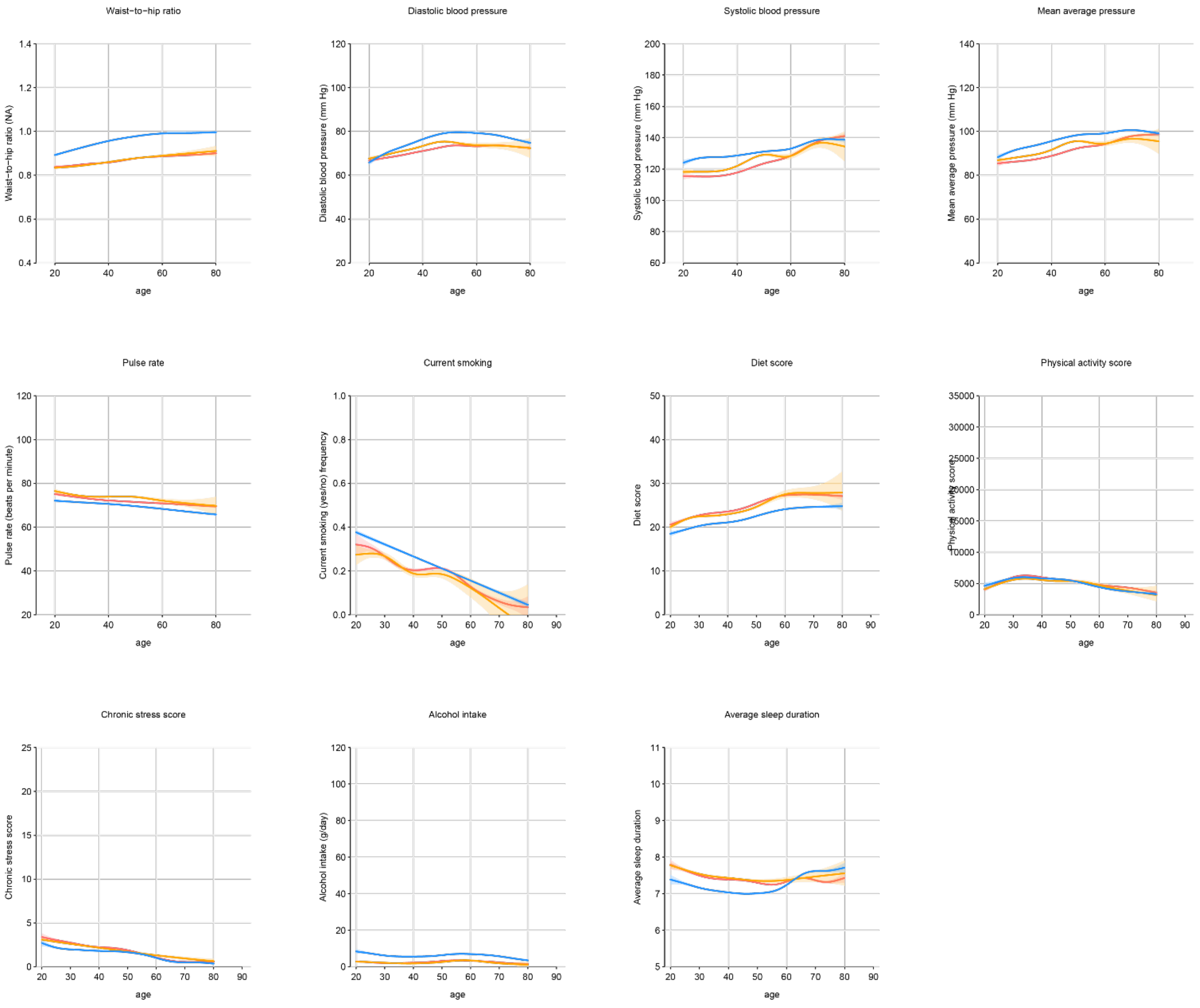

Supplementary Figure 3. The effect of hormone therapy on age-dependent sex differences in 51 phenotypes.

For each phenotype, GAM-based fitted lines are plotted for men (blue) and women not taking hormone medications (red) and for women taking hormone medications (yellow). Confidence intervals (+/- 1.96 SE) are depicted around the lines in a more transparent hue. Plots are given for all phenotypes without multiple testing correction.

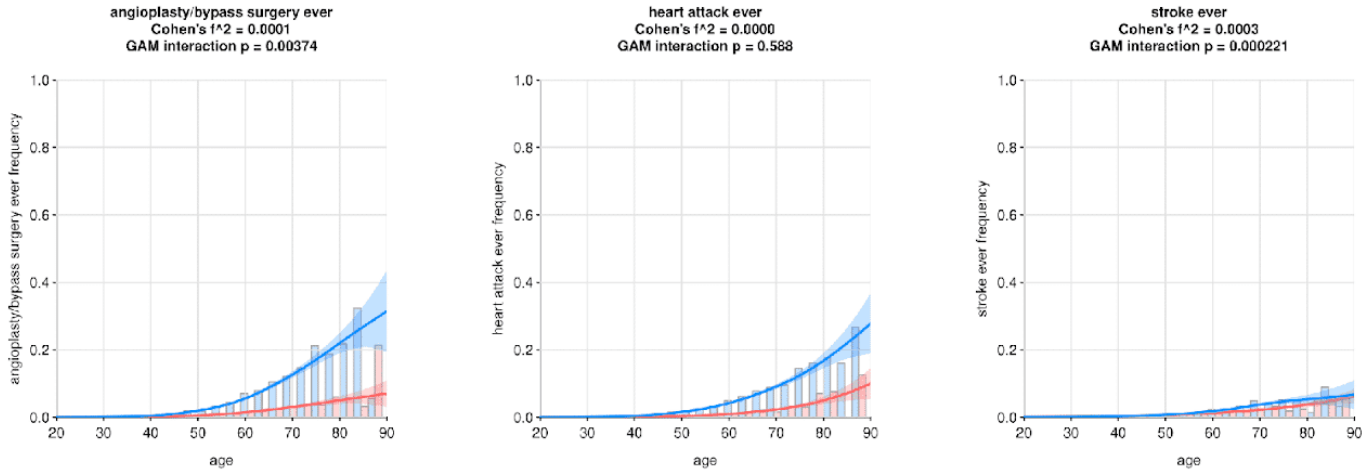

Supplementary Figure 4. Age-dependent sex differences in CVDs.

For each phenotype, the plot shows bars representing phenotype frequency for each 3 years of age and fitted lines obtained using GAM plotted: men (blue) and women (red). Confidence intervals (+/- 1.96 SE) around the lines are depicted in a more transparent hue. Cohen's f^2 reflects the effect size of the age x sex interaction. GAM interaction P represents the significance of the age by sex interaction term. Plots are given for all phenotypes without multiple testing correction.

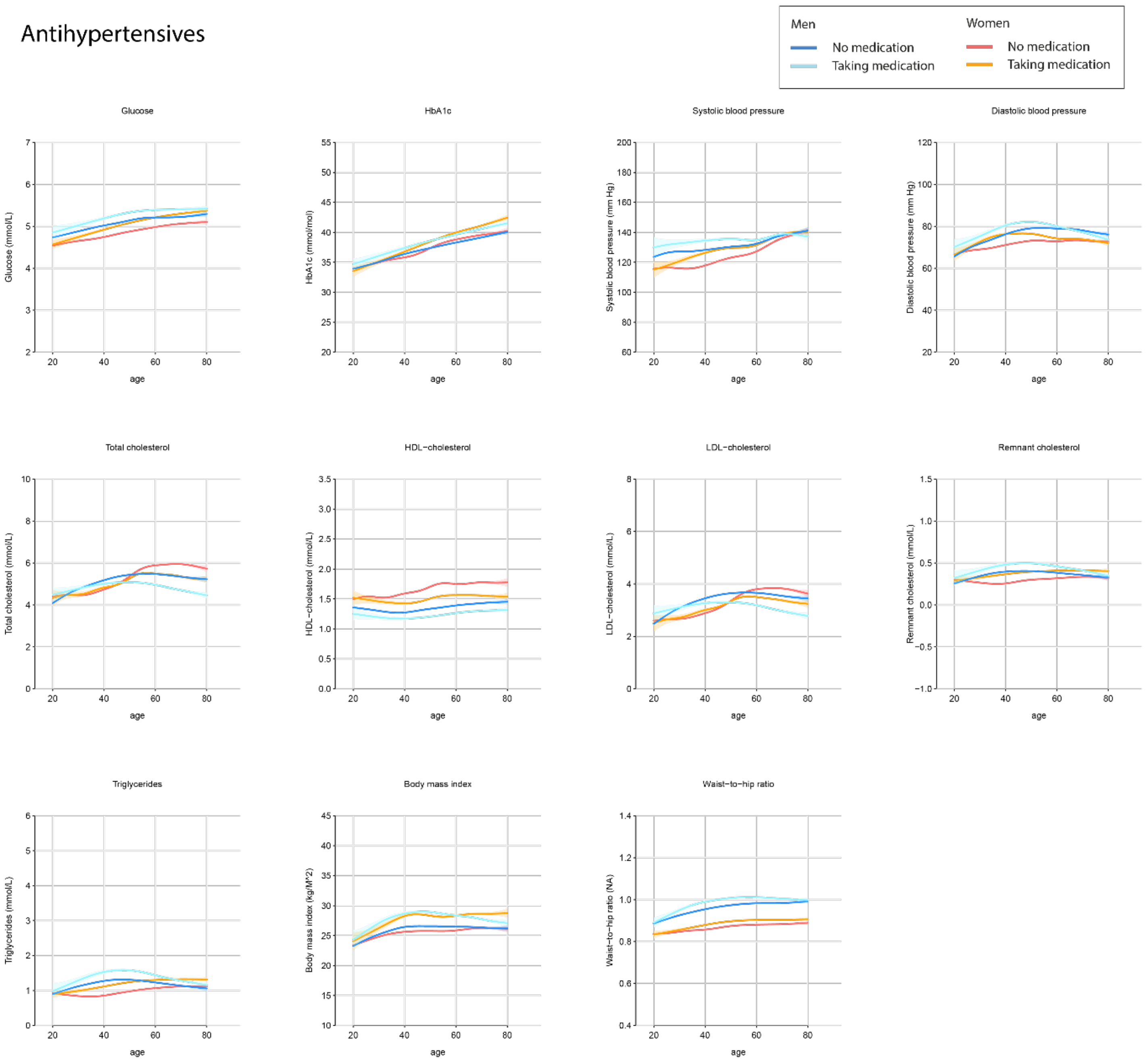

Supplementary Figure 5. The effect of antihypertensive medication usage on the age-dependent sex differences in CMD risk factors

For each CMD-related phenotype, GAM-based fitted lines are plotted for men not taking antihypertensive medication (blue), men taking antihypertensive medication (cyan), women not taking antihypertensive medications (red) and women taking antihypertensive medications (yellow). Confidence intervals (+/- 1.96 SE) are depicted around the lines in a more transparent hue.

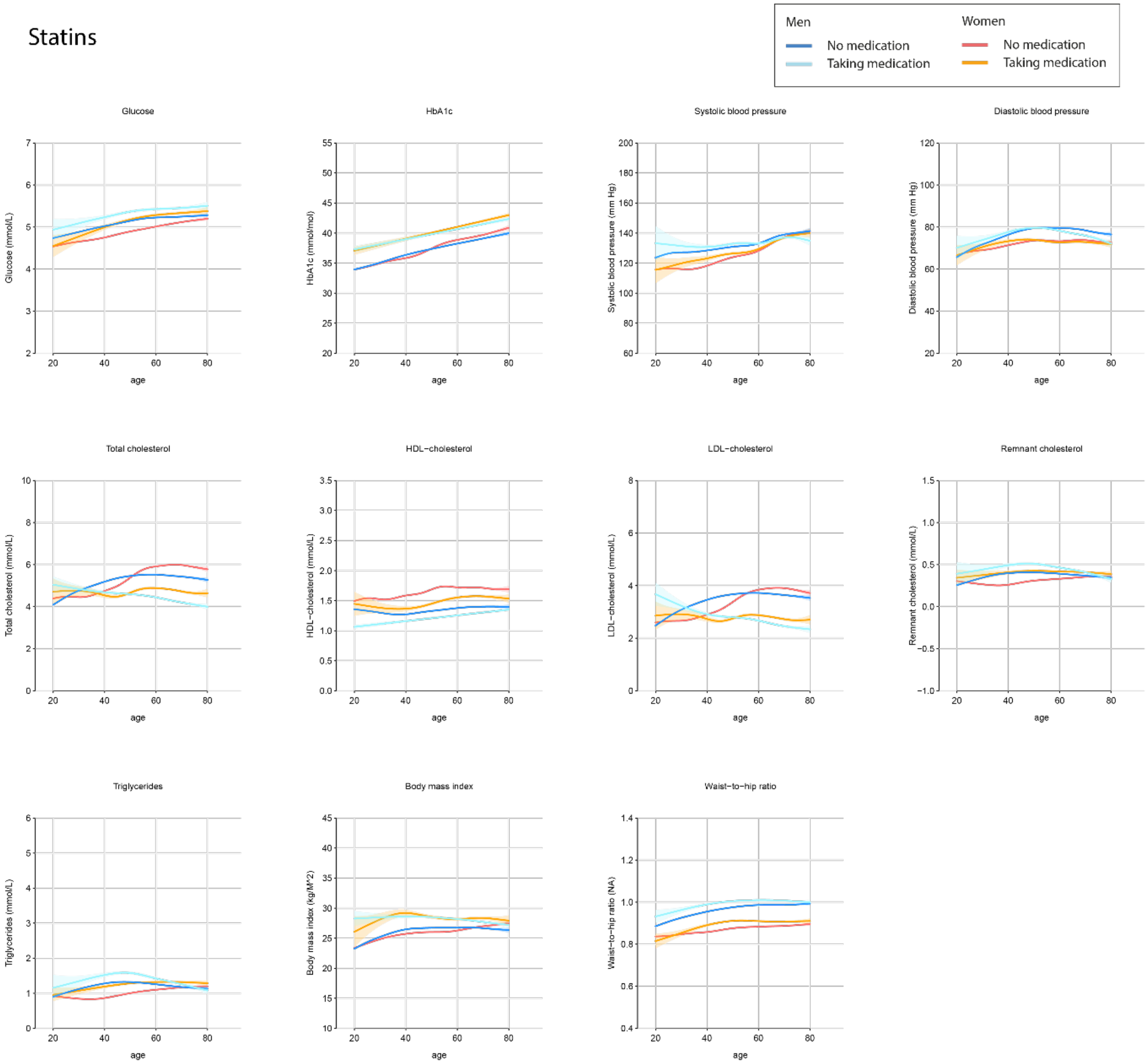

Supplementary Figure 6. The effect of statin medication usage on age-dependent sex differences in CMD risk factors

For each CMD-related phenotype, GAM-based fitted lines are plotted for men not taking statins (blue), men taking statins (cyan), women not taking statins (red) and women taking statins (yellow). Confidence intervals (+/- 1.96 SE) are depicted around the lines in a more transparent hue.

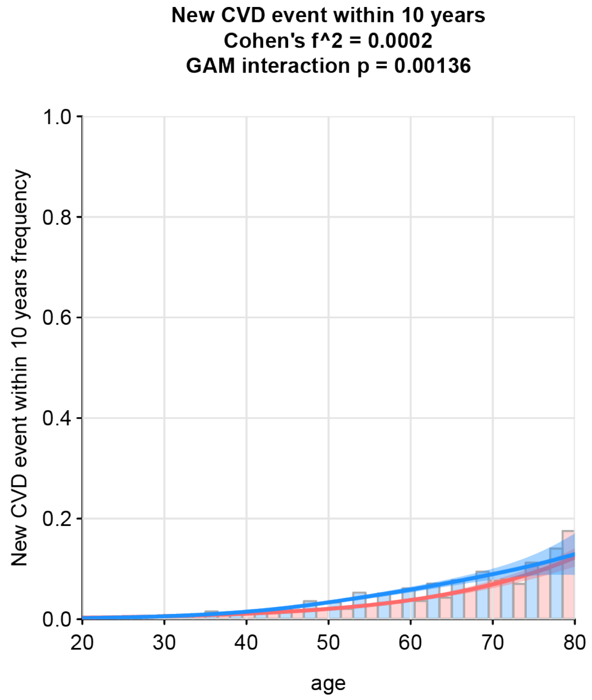

Supplementary Figure 7. Age-dependent sex differences in new CVD cases within 10 years after baseline visit. The plot shows bars representing new CVD occurrence frequency for each 3 years of age at baseline and fitted lines obtained using GAM: men (blue) and women (red). Confidence intervals (+/- 1.96 SE) around the lines are depicted in a more transparent hue. Cohen's f^2 reflects the effect size of the age x sex interaction. GAM interaction P represents the significance of the age by sex interaction term.

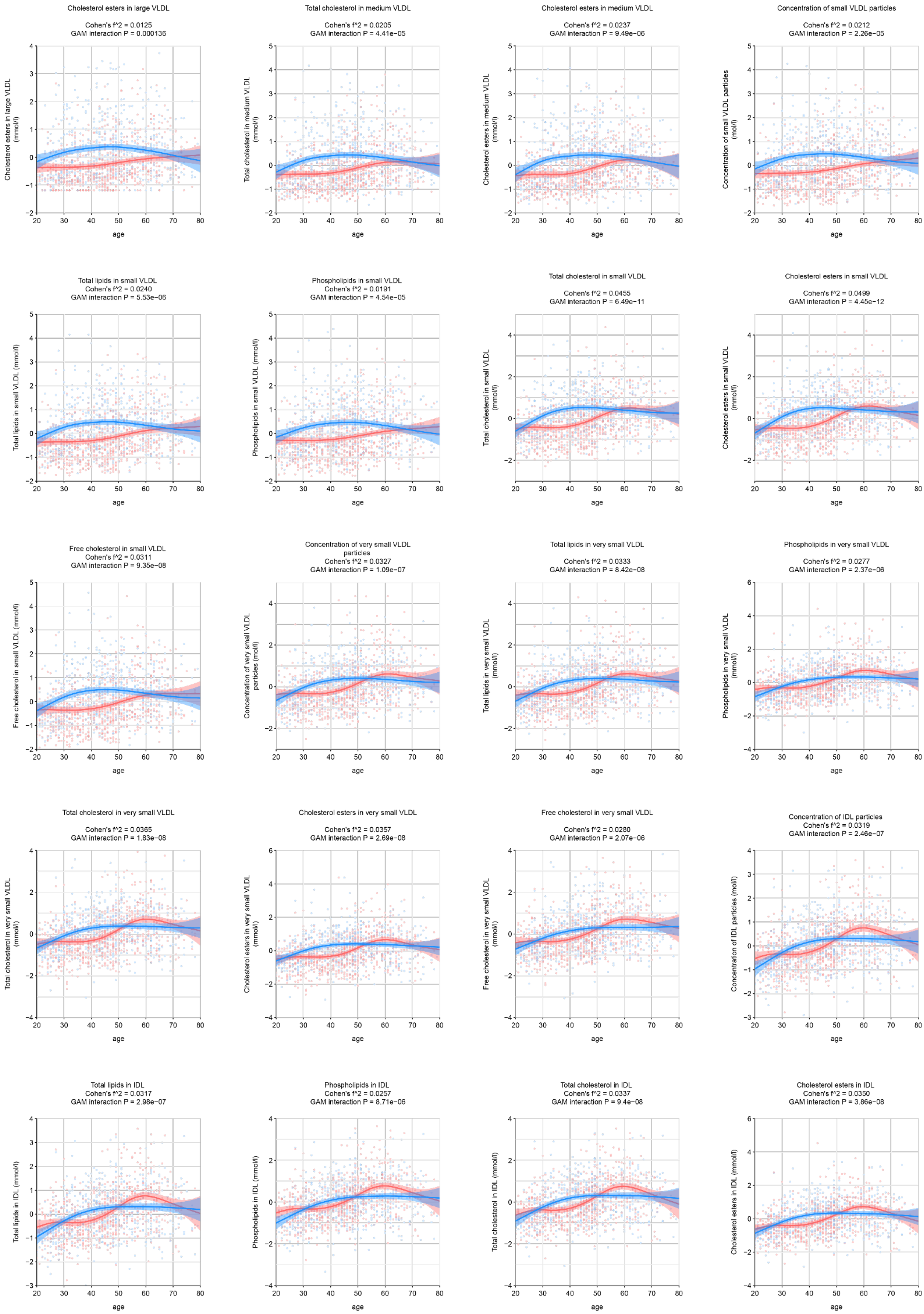
Cholesterol esters in large VLDL
Cohen's f^2 = 0.0125
GAM interaction P = 0.000136
Cholesterol esters in large VLDL (mmol/l)
age
Total cholesterol in medium VLDL
Cohen's f^2 = 0.0205
GAM interaction P = 4.41e−05
Total cholesterol in medium VLDL (mmol/l)
age
Cholesterol esters in medium VLDL
Cohen's f^2 = 0.0237
GAM interaction P = 9.49e−06
Cholesterol esters in medium VLDL (mmol/l)
age
Concentration of small VLDL particles
Cohen's f^2 = 0.0212
GAM interaction P = 2.26e−05
Concentration of small VLDL particles (mol/l)
age
Total lipids in small VLDL
Cohen's f^2 = 0.0240
GAM interaction P = 5.53e−06
Total lipids in small VLDL (mmol/l)
age
Phospholipids in small VLDL
Cohen's f^2 = 0.0191
GAM interaction P = 4.54e−05
Phospholipids in small VLDL (mmol/l)
age
Total cholesterol in small VLDL
Cohen's f^2 = 0.0455
GAM interaction P = 6.49e−11
Total cholesterol in small VLDL (mmol/l)
age
Cholesterol esters in small VLDL
Cohen's f^2 = 0.0499
GAM interaction P = 4.45e−12
Cholesterol esters in small VLDL (mmol/l)
age
Free cholesterol in small VLDL
Cohen's f^2 = 0.0311
GAM interaction P = 9.35e−08
Free cholesterol in small VLDL (mmol/l)
age
Concentration of very small VLDL particles
Cohen's f^2 = 0.0327
GAM interaction P = 1.09e−07
Concentration of very small VLDL particles (mol/l)
age
Total lipids in very small VLDL
Cohen's f^2 = 0.0333
GAM interaction P = 8.42e−08
Total lipids in very small VLDL (mmol/l)
age
Phospholipids in very small VLDL
Cohen's f^2 = 0.0277
GAM interaction P = 2.37e−06
Phospholipids in very small VLDL (mmol/l)
age
Total cholesterol in very small VLDL
Cohen's f^2 = 0.0365
GAM interaction P = 1.83e−08
Total cholesterol in very small VLDL (mmol/l)
age
Cholesterol esters in very small VLDL
Cohen's f^2 = 0.0357
GAM interaction P = 2.69e−08
Cholesterol esters in very small VLDL (mmol/l)
age
Free cholesterol in very small VLDL
Cohen's f^2 = 0.0280
GAM interaction P = 2.07e−06
Free cholesterol in very small VLDL (mmol/l)
age
Concentration of IDL particles
Cohen's f^2 = 0.0319
GAM interaction P = 2.46e−07
Concentration of IDL particles (mol/l)
age
Total lipids in IDL
Cohen's f^2 = 0.0317
GAM interaction P = 2.98e−07
Total lipids in IDL (mmol/l)
age
Phospholipids in IDL
Cohen's f^2 = 0.0257
GAM interaction P = 8.71e−06
Phospholipids in IDL (mmol/l)
age
Total cholesterol in IDL
Cohen's f^2 = 0.0337
GAM interaction P = 9.4e−08
Total cholesterol in IDL (mmol/l)
age
Cholesterol esters in IDL
Cohen's f^2 = 0.0350
GAM interaction P = 3.86e−08
Cholesterol esters in IDL (mmol/l)
age

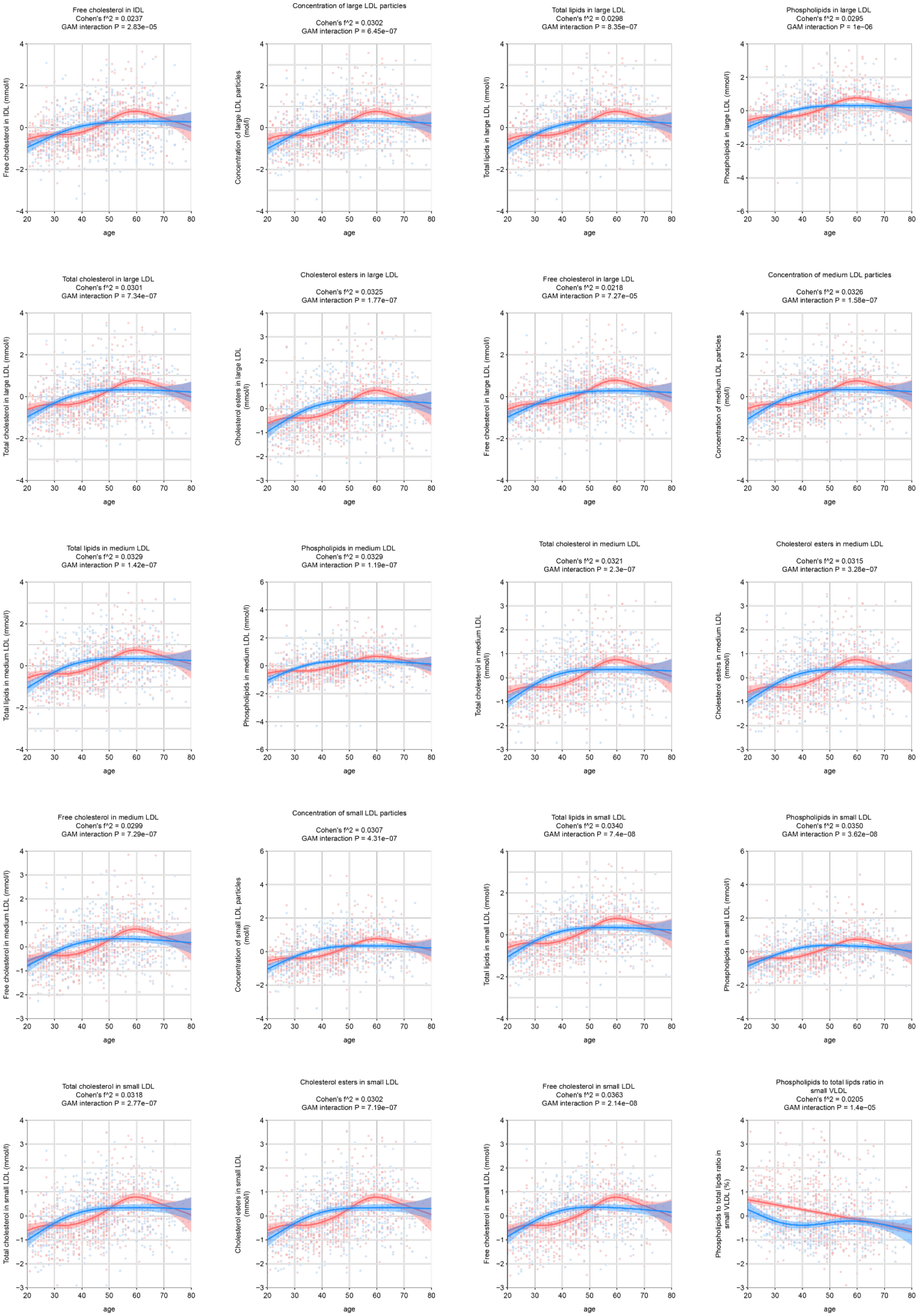
Free cholesterol in IDL
Cohen's f^2 = 0.0237
GAM interaction P = 2.83e−05
Free cholesterol in IDL (mmol/l)
age
Concentration of large LDL particles
Cohen's f^2 = 0.0302
GAM interaction P = 6.45e−07
Concentration of large LDL particles (mol/l)
age
Total lipids in large LDL
Cohen's f^2 = 0.0298
GAM interaction P = 8.35e−07
Total lipids in large LDL (mmol/l)
age
Phospholipids in large LDL
Cohen's f^2 = 0.0295
GAM interaction P = 1e−06
Phospholipids in large LDL (mmol/l)
age
Total cholesterol in large LDL
Cohen's f^2 = 0.0301
GAM interaction P = 7.34e−07
Total cholesterol in large LDL (mmol/l)
age
Cholesterol esters in large LDL
Cohen's f^2 = 0.0325
GAM interaction P = 1.77e−07
Cholesterol esters in large LDL (mmol/l)
age
Free cholesterol in large LDL
Cohen's f^2 = 0.0218
GAM interaction P = 7.27e−05
Free cholesterol in large LDL (mmol/l)
age
Concentration of medium LDL particles
Cohen's f^2 = 0.0326
GAM interaction P = 1.58e−07
Concentration of medium LDL particles (mol/l)
age
Total lipids in medium LDL
Cohen's f^2 = 0.0329
GAM interaction P = 1.42e−07
Total lipids in medium LDL (mmol/l)
age
Phospholipids in medium LDL
Cohen's f^2 = 0.0329
GAM interaction P = 1.19e−07
Phospholipids in medium LDL (mmol/l)
age
Total cholesterol in medium LDL
Cohen's f^2 = 0.0321
GAM interaction P = 2.3e−07
Total cholesterol in medium LDL (mmol/l)
age
Cholesterol esters in medium LDL
Cohen's f^2 = 0.0315
GAM interaction P = 3.28e−07
Cholesterol esters in medium LDL (mmol/l)
age
Free cholesterol in medium LDL
Cohen's f^2 = 0.0299
GAM interaction P = 7.29e−07
Free cholesterol in medium LDL (mmol/l)
age
Concentration of small LDL particles
Cohen's f^2 = 0.0307
GAM interaction P = 4.31e−07
Concentration of small LDL particles (mol/l)
age
Total lipids in small LDL
Cohen's f^2 = 0.0340
GAM interaction P = 7.4e−08
Total lipids in small LDL (mmol/l)
age
Phospholipids in small LDL
Cohen's f^2 = 0.0350
GAM interaction P = 3.62e−08
Phospholipids in small LDL (mmol/l)
age
Total cholesterol in small LDL
Cohen's f^2 = 0.0318
GAM interaction P = 2.77e−07
Total cholesterol in small LDL (mmol/l)
age
Cholesterol esters in small LDL
Cohen's f^2 = 0.0302
GAM interaction P = 7.19e−07
Cholesterol esters in small LDL (mmol/l)
age
Free cholesterol in small LDL
Cohen's f^2 = 0.0363
GAM interaction P = 2.14e−08
Free cholesterol in small LDL (mmol/l)
age
Phospholipids to total lipds ratio in small VLDL
Cohen's f^2 = 0.0205
GAM interaction P = 1.4e−05
Phospholipids to total lipids ratio in small VLDL (%)
age

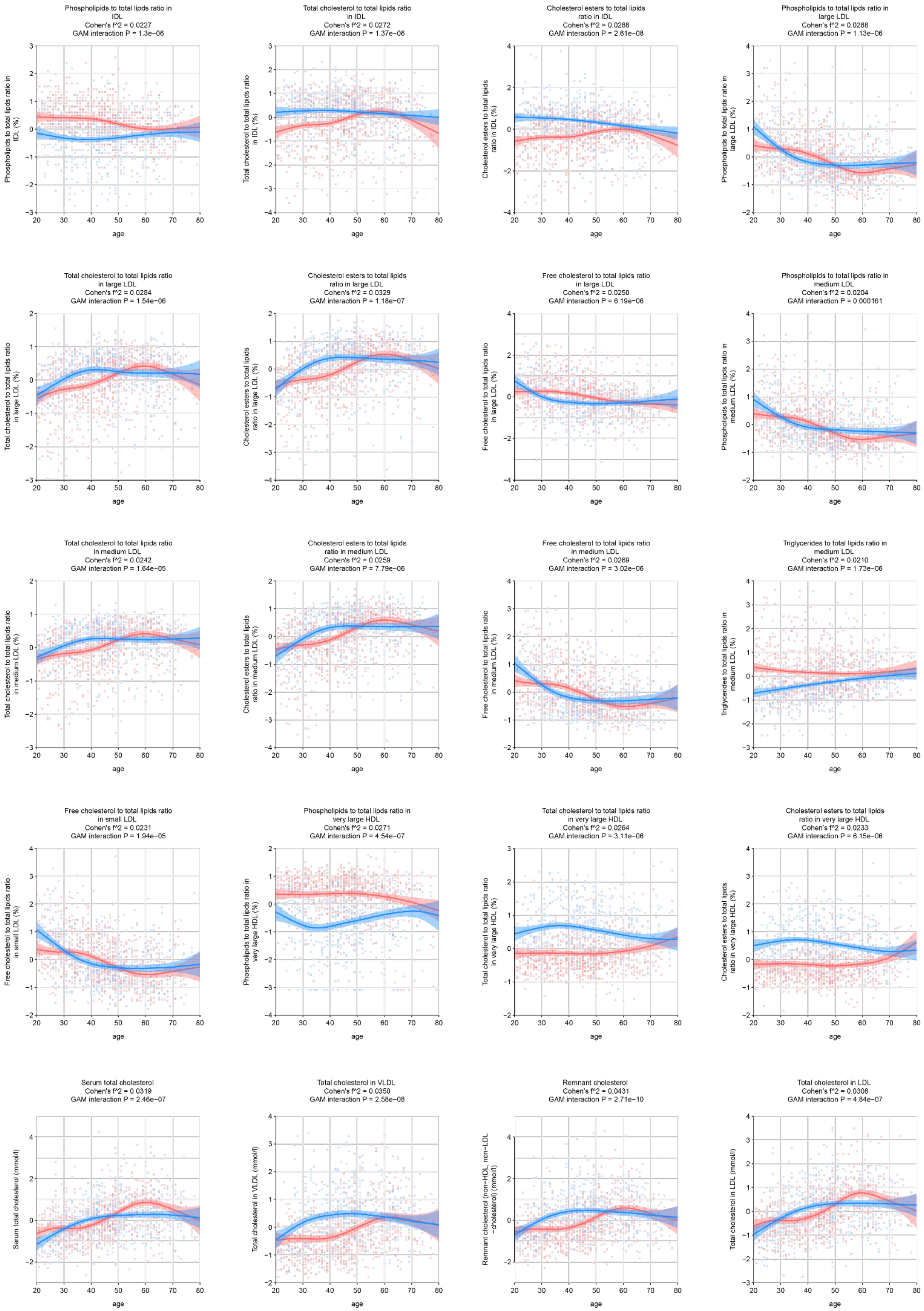

Phospholipids to total lipids ratio in IDL
Cohen's f^2 = 0.0227
GAM interaction P = 1.3e−06
Phospholipids to total lipids ratio in IDL (%)
Total cholesterol to total lipids ratio in IDL
Cohen's f^2 = 0.0272
GAM interaction P = 1.37e−06
Total cholesterol to total lipids ratio in IDL (%)
Cholesterol esters to total lipids ratio in IDL
Cohen's f^2 = 0.0288
GAM interaction P = 2.61e−08
Cholesterol esters to total lipids ratio in IDL (%)
Phospholipids to total lipds ratio in large LDL
Cohen's f^2 = 0.0288
GAM interaction P = 1.13e−06
Phospholipids to total lipids ratio in large LDL (%)
Total cholesterol to total lipids ratio in large LDL
Cohen's f^2 = 0.0284
GAM interaction P = 1.54e−06
Total cholesterol to total lipids ratio in large LDL (%)
Cholesterol esters to total lipids ratio in large LDL
Cohen's f^2 = 0.0329
GAM interaction P = 1.18e−07
Cholesterol esters to total lipids ratio in large LDL (%)
Free cholesterol to total lipids ratio in large LDL
Cohen's f^2 = 0.0250
GAM interaction P = 6.19e−06
Free cholesterol to total lipids ratio in large LDL (%)
Phospholipids to total lipds ratio in medium LDL
Cohen's f^2 = 0.0204
GAM interaction P = 0.000161
Phospholipids to total lipids ratio in medium LDL (%)
Total cholesterol to total lipids ratio in medium LDL
Cohen's f^2 = 0.0242
GAM interaction P = 1.64e−05
Total cholesterol to total lipids ratio in medium LDL (%)
Cholesterol esters to total lipids ratio in medium LDL
Cohen's f^2 = 0.0259
GAM interaction P = 7.79e−06
Cholesterol esters to total lipids ratio in medium LDL (%)
Free cholesterol to total lipids ratio in medium LDL
Cohen's f^2 = 0.0269
GAM interaction P = 3.02e−06
Free cholesterol to total lipids ratio in medium LDL (%)
Triglycerides to total lipids ratio in medium LDL
Cohen's f^2 = 0.0210
GAM interaction P = 1.73e−06
Triglycerides to total lipids ratio in medium LDL (%)
Free cholesterol to total lipids ratio in small LDL
Cohen's f^2 = 0.0231
GAM interaction P = 1.94e−05
Free cholesterol to total lipids ratio in small LDL (%)
Phospholipids to total lipds ratio in very large HDL
Cohen's f^2 = 0.0271
GAM interaction P = 4.54e−07
Phospholipids to total lipds ratio in very large HDL (%)
Total cholesterol to total lipids ratio in very large HDL
Cohen's f^2 = 0.0264
GAM interaction P = 3.11e−06
Total cholesterol to total lipids ratio in very large HDL (%)
Cholesterol esters to total lipids ratio in very large HDL
Cohen's f^2 = 0.0233
GAM interaction P = 6.15e−06
Cholesterol esters to total lipids ratio in very large HDL (%)
Serum total cholesterol
Cohen's f^2 = 0.0319
GAM interaction P = 2.46e−07
Serum total cholesterol (mmol/l)
Total cholesterol in VLDL
Cohen's f^2 = 0.0350
GAM interaction P = 2.58e−08
Total cholesterol in VLDL (mmol/l)
Remnant cholesterol
Cohen's f^2 = 0.0431
GAM interaction P = 2.71e−10
Remnant cholesterol (non−HDL, non−LDL −cholesterol) (mmol/l)
Total cholesterol in LDL
Cohen's f^2 = 0.0308
GAM interaction P = 4.84e−07
Total cholesterol in LDL (mmol/l)
age

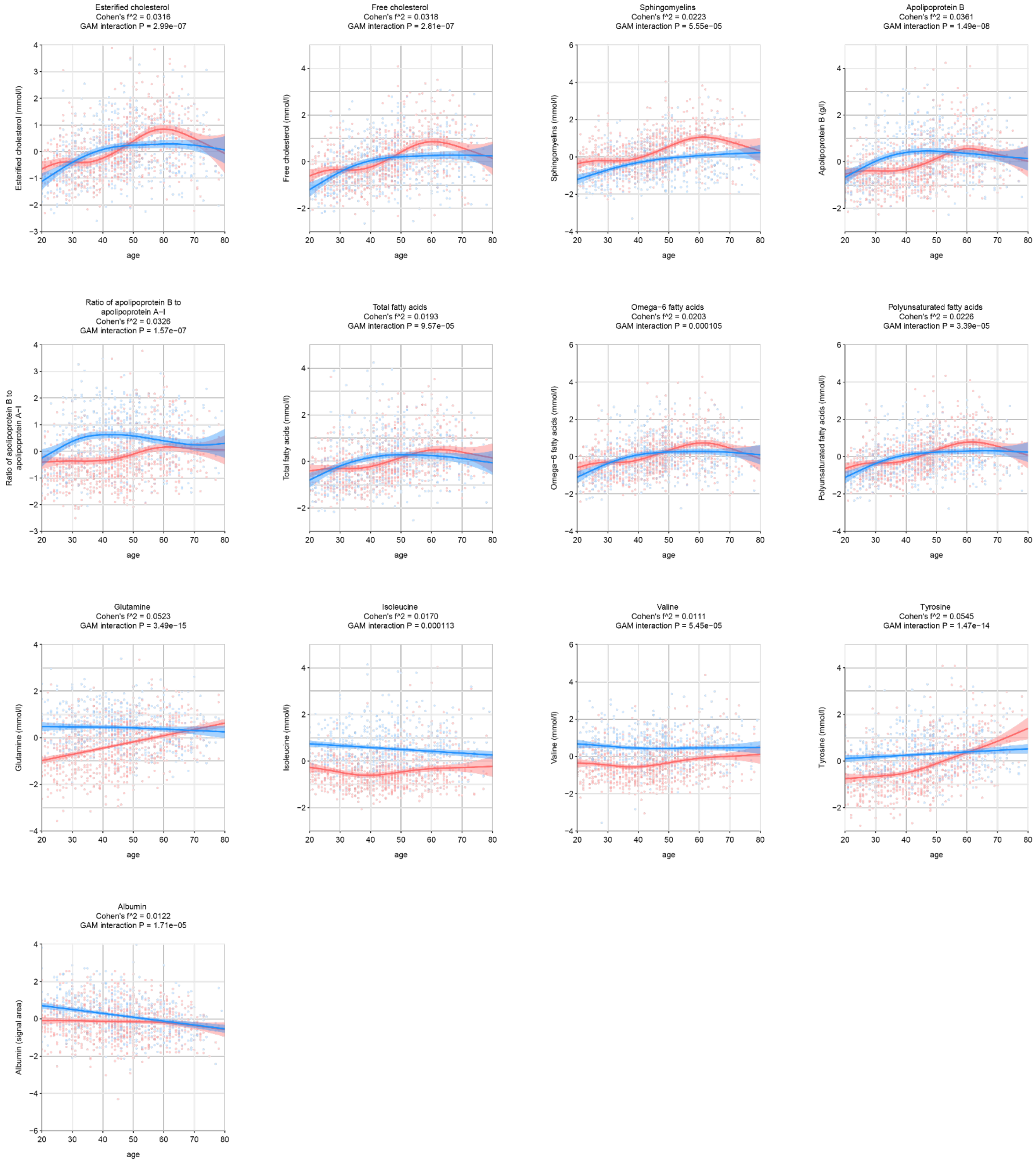

Supplementary Figure 8. Age-dependent sex differences for NMR-based lipid and lipoprotein levels.

For each metabolite, plots show scaled values per individual and fitted lines obtained using GAM: men (blue) and women (red). Confidence intervals (+/- 1.96 SE) around the lines are depicted using a more transparent hue. F^2 (Cohen's f^2) reflects the effect size of the age by sex interaction. GAM interaction p indicates the significance of the age by sex interaction term. Only results with a significant age by sex interaction after Bonferroni correction are plotted.
