# Supplementary material for: Age-dependent sex differences in cardiometabolic risk factors
Source: Nat Cardiovasc Res. 2022 Sep 12;1(9):844–54. doi: 10.1038/s44161-022-00131-8 (PMC11357998; doi:10.1038/s44161-022-00131-8)
Supplement: Supplementary file 2 — Reporting Summary [file 44161_2022_131_MOESM2_ESM.pdf]

nature portfolio

Corresponding author(s): Daria V. Zhernakova

Last updated by author(s): Aug 3, 2022

Reporting Summary

Nature Portfolio wishes to improve the reproducibility of the work that we publish. This form provides structure for consistency and transparency in reporting. For further information on Nature Portfolio policies, see our Editorial Policies and the Editorial Policy Checklist.

Statistics

For all statistical analyses, confirm that the following items are present in the figure legend, table legend, main text, or Methods section.

n/a	Confirmed	
☐	☒	The exact sample size (*n*) for each experimental group/condition, given as a discrete number and unit of measurement
☐	☒	A statement on whether measurements were taken from distinct samples or whether the same sample was measured repeatedly
☐	☒	The statistical test(s) used AND whether they are one- or two-sided<br>*Only common tests should be described solely by name; describe more complex techniques in the Methods section.*
☐	☒	A description of all covariates tested
☐	☒	A description of any assumptions or corrections, such as tests of normality and adjustment for multiple comparisons
☐	☒	A full description of the statistical parameters including central tendency (e.g. means) or other basic estimates (e.g. regression coefficient) AND variation (e.g. standard deviation) or associated estimates of uncertainty (e.g. confidence intervals)
☐	☒	For null hypothesis testing, the test statistic (e.g. *F*, *t*, *r*) with confidence intervals, effect sizes, degrees of freedom and *P* value noted<br>*Give P values as exact values whenever suitable.*
☒	☐	For Bayesian analysis, information on the choice of priors and Markov chain Monte Carlo settings
☒	☐	For hierarchical and complex designs, identification of the appropriate level for tests and full reporting of outcomes
☐	☒	Estimates of effect sizes (e.g. Cohen's *d*, Pearson's *r*), indicating how they were calculated

Our web collection on statistics for biologists contains articles on many of the points above.

Software and code

Policy information about availability of computer code

Data collection	No software was used for data collection
Data analysis	All data analyses were conducted using publicly available tools. Data analysis was performed in R using the following R packages: mgcv (v. 1.8-31), gamclass (v. 0.62.3), vegan (v. 2.5-6), circlize (v. 0.4.14) and custom scripts publicly available at: https://github.com/DashaZhernakova/umcg_scripts/tree/master/age_sex_interactions_paper

For manuscripts utilizing custom algorithms or software that are central to the research but not yet described in published literature, software must be made available to editors and reviewers. We strongly encourage code deposition in a community repository (e.g. GitHub). See the Nature Portfolio guidelines for submitting code & software for further information.

Data

Policy information about availability of data

All manuscripts must include a data availability statement. This statement should provide the following information, where applicable:

- Accession codes, unique identifiers, or web links for publicly available datasets
- A description of any restrictions on data availability
- For clinical datasets or third party data, please ensure that the statement adheres to our policy

Participant phenotype data are not publicly available to protect participants’ privacy. These data can be requested by sending a scientific proposal to the Lifelines

Biobank (https://www.lifelines.nl/researcher/how-to-apply). All data access to the Lifelines population cohort must follow the informed consent regulations of the Medical Ethics Review Board of the University Medical Center Groningen described at http://lifelines.nl/.

Human research participants

Policy information about studies involving human research participants and Sex and Gender in Research.

Reporting on sex and gender	The aim of this paper was to study age effect on sex differences in clinical and molecular phenotypes, thus all analyses were sex-based.
Population characteristics	Our study involved 146,021 individuals (58% female) across an age span from 20 to 80 years from the Dutch population-based cohort Lifelines. The average age of participants was 45 years old (SD = 12).
Recruitment	Participants of the Lifelines cohort have been recruited in three stages: recruitment of an index population via general practitioners, subsequent inclusion of their family members, and online self-registration. This cohort is considered representative of adult population of the North of the Netherlands. Thus some of the reported results may be population-specific and not generalizable to non-European ethnicities. Another potential bias is that CVD status was self-reported, which likely leads to underestimation of disease incidence and the number of new CVD events, especially for women. In addition, this study only included individuals between 20 and 80 years of age, thus the sex differences in childhood and older age could not be estimated.
Ethics oversight	The Lifelines study was approved by the ethics committee of the University Medical Center Groningen, document number METc2007/152. All Lifelines participants signed an informed consent form prior to sample collection.

Note that full information on the approval of the study protocol must also be provided in the manuscript.

Field-specific reporting

Please select the one below that is the best fit for your research. If you are not sure, read the appropriate sections before making your selection.

☒ Life sciences ☐ Behavioural & social sciences ☐ Ecological, evolutionary & environmental sciences

For a reference copy of the document with all sections, see nature.com/documents/nr-reporting-summary-flat.pdf

Life sciences study design

All studies must disclose on these points even when the disclosure is negative.

Sample size	Our study involved 146,021 individuals from the Dutch population-based cohort Lifelines. For a subset of this cohort additional data were examined: plasma proteomics was available for 1,447 samples and NMR lipidomics was available for 1,440 samples. In order to ensure the analysis power, our study includes as many samples as possible, thus no sample size calculation was performed.
Data exclusions	In this study we selected participants across an age span from 20 to 80 years due to low sample size outside this range. To meet the requirements of generalized additive models we removed extreme outliers by excluding observations that were more than three interquartile ranges below the first quartile or more than three interquartile ranges above the third quartile from the whole dataset, not taking age and sex into account. We report both the results without covariate correction and the results corrected for current smoking, hormone therapy,BMI, glucose levels, total cholesterol, HDL cholesterol, systolic blood pressure and type 2 diabetes.
Replication	To ensure the reproducibility of our results we performed 10-fold cross-validation and bootstrapping.
Randomization	This is human cohort-based analysis. The sample collection and sequencing were performed in a random order. No extra randomization was done for this study.
Blinding	This study is a human cohort-based, observational study. Thus no blinding was performed.

Reporting for specific materials, systems and methods

We require information from authors about some types of materials, experimental systems and methods used in many studies. Here, indicate whether each material, system or method listed is relevant to your study. If you are not sure if a list item applies to your research, read the appropriate section before selecting a response.

Materials & experimental systems

n/a	Involved in the study
☒	☐ Antibodies
☒	☐ Eukaryotic cell lines
☒	☐ Palaeontology and archaeology
☒	☐ Animals and other organisms
☒	☐ Clinical data
☒	☐ Dual use research of concern

Methods

n/a	Involved in the study
☒	☐ ChIP-seq
☒	☐ Flow cytometry
☒	☐ MRI-based neuroimaging
